# The functions and factors governing fungal communities and diversity in agricultural waters: insights into the ecosystem services aquatic mycobiota provide

**DOI:** 10.3389/fmicb.2024.1460330

**Published:** 2024-11-05

**Authors:** Phillip Pham, Yichao Shi, Izhar Khan, Mark Sumarah, Justin Renaud, Mark Sunohara, Emilia Craiovan, David Lapen, Stéphane Aris-Brosou, Wen Chen

**Affiliations:** ^1^Ottawa Research and Development Centre, Science and Technology Branch, Agriculture and Agri-Food Canada, Ottawa, ON, Canada; ^2^Department of Biology, University of Ottawa, Ottawa, ON, Canada; ^3^London Research Development Centre, Science and Technology Branch, Agriculture and Agri-Food Canada, London, ON, Canada; ^4^Department of Mathematics and Statistics, University of Ottawa, Ottawa, ON, Canada

**Keywords:** aquatic fungi, metabarcoding, community ecology, land use, agriculture

## Abstract

**Introduction:**

Fungi are essential to the aquatic food web, nutrient cycling, energy flow, and ecosystem regulation. Fungal community structures in water can be influenced by adjacent terrestrial environments, which drive and control some ecosystem services they provide. However, the roles of freshwater fungal communities remain underexplored compared to bacterial communities in this context.

**Methods:**

We assessed the impact of anthropological and environmental factors on freshwater mycobiota in an agriculturally dominated water basin in eastern Ontario, Canada. We undertook bi-weekly surface water sampling from 2016 to 2021 and conducted fungal internal transcribed spacer 2 (ITS2) metabarcoding on the samples, complemented by ancillary data, including water physicochemical properties, upstream land use, hydrology, and weather conditions.

**Results:**

Our study yielded 6,571 OTUs from 503 water samples, spanning 15 fungal phyla, dominated by Ascomycota, Basidiomycota, and Chytridiomycota. Agricultural land use was associated with decreased mycobiota alpha diversity and distinct fungal communities were observed at agricultural drainage ditch and mixed-land use sites compared to a forested site that had minimal anthropogenic activities in catchment. Notably, river discharge emerged as a predominant influencer of both community diversity and composition, likely amplified by precipitation-induced erosion and drainage from adjacent terrestrial environments.

**Discussion:**

Water physicochemical properties, including stream fungicide levels, explained a small proportion of the variation in mycobiota communities, underscoring the significance of unmeasured factors, alongside stochastic community assembly processes. Nevertheless, stream mycobiota demonstrated functional resilience for critical ecological processes under different environmental conditions. Altogether, these results highlight the complex interplay of factors influencing the freshwater mycobiota, which is essential for elevated understanding of the ecosystem services these fungi provide.

## 1 Introduction

Fungi in aquatic environments are recognized for their role in decomposing allochthonous organic matter, transforming it into nutrients accessible to other trophic levels in the freshwater food web, such as zooplankton and bacteria (Krauss et al., 2011; Bärlocher, 2016; Grossart et al., 2019). Despite their important role, less than 10% of the estimated 1.5 million fungal species on Earth have been characterized, with just 3,000–4,000 known true aquatic fungi (Ittner et al., 2018; Lepere et al., 2019; Calabon et al., 2023). The low number of identified aquatic fungal species is partly due to the challenges in culturing them and the limitations of traditional *in situ* morphological characterization methods (Bärlocher, 2016). Recent advances in high-throughput sequencing (HTS) and phenotyping technologies have uncovered a multitude of potentially new fungal species found in aquatic environments, often referred to as “dark matter fungi” (Grossart et al., 2016). For instance, a study by Lepere et al. (2019) using 18S rRNA gene metabarcoding data from 25 lakes and four rivers in Europe, the Arctic, and the Himalayas, uncovered over 25,000 operational taxonomic units (OTUs), each representing a putative fungal species. Their study demonstrated that the number of recovered putative fungal species in these freshwater systems alone is eight times greater than the number of known aquatic fungal species. This underscores the widespread underestimation and limited characterization of fungal diversity in aquatic environments (Grossart et al., 2016; Grossart and Rojas-Jimenez, 2016), notwithstanding the understanding of their specific functions. It is worth noting that “freshwater fungal communities” recovered through HTS of environmental DNA encompass both strictly aquatic fungi, which complete part or all of their life cycles underwater (Shearer et al., 2007; Grossart et al., 2019), and transient terrestrial fungi entering from external environments.

Given the important roles of aquatic fungi in nutrient cycling and bioremediation (Seena et al., 2022), it is critical to identify environmental and anthropogenic factors that govern and regulate fungal biodiversity (Duarte et al., 2006; Duarte et al., 2008). Like other microbial members found in aquatic ecosystems, the aquatic fungal communities are influenced by mass exchange with adjacent terrestrial environments and water physical, chemical, and biological properties (Krauss et al., 2011; Ittner et al., 2018). For instance, runoff and drainage from agricultural lands can significantly influence surface water quality (e.g., agro-chemicals, sediment, nutrients) and hydrology (Carpenter et al., 1998; Kato et al., 2009), and consequently alter the diversity and composition of freshwater mycobiota (Chen et al., 2018; Baudy et al., 2021). Previous studies have shown that the biomass of aquatic fungi and leaf decomposition rates increased under elevated nitrogen and phosphorus levels (Gulis and Suberkropp, 2003; Biasi et al., 2017). However, in other situations, high concentrations of nitrogen and metals have been shown to decrease decomposition rates, particularly when there is a decline in dissolved oxygen (Solé et al., 2008; Bruder et al., 2016). Collectively, freshwater fungal community heterogeneity was found to be influenced in particular by water flow rates, water pH and temperature, dissolved organic matter, nitrogen, phosphorus, oxygen, sulfate, and chloride ions (Bai et al., 2018; Ortiz-Vera et al., 2018; Pietryczuk et al., 2018). However, limited research has been conducted on how agricultural and other land use activities impact the mycobiota in freshwater systems (Bai et al., 2018; Ittner et al., 2018; Ortiz-Vera et al., 2018; Baudy et al., 2021). In this context, runoff and drainage from agricultural land can introduce detectable quantities of fungicides (Zubrod et al., 2019) and allochthonous fungal species, leading to shifts in fungal diversity and community composition in affected water bodies (Chauvet et al., 2016; Baudy et al., 2021; Ma et al., 2021). Notably, previous studies have shown that agricultural practices can alter the composition of freshwater fungal communities but induce functional redundancy, allowing them to maintain their ecological roles, such as leaf litter decomposition, despite environmental changes (Martínez et al., 2020; Baudy et al., 2021). However, our understanding of the diversity and ecological roles of fungal communities in water under agricultural land uses remains limited compared to the extensive research that has been conducted on invertebrates and bacteria in aquatic ecosystems (Keck et al., 2017; Pawlowski et al., 2018; Bush et al., 2019; Sagova-Mareckova et al., 2021).

This study aimed to deepen the understanding of the diversity, composition, and functionality of freshwater fungal communities influenced by agricultural land use. We sought to characterize their responses to environmental factors, especially the nature of upstream land use activities. We hypothesized that agricultural land use exerts selective pressure on stream mycobiota, altering diversity and taxonomic composition while maintaining ecological functionality. To test this, we performed bi-weekly surface water sampling in an agriculturally dominated water basin and characterized the fungal communities using ITS2 metabarcoding of the fungal rRNA gene. Sample-associated metadata included upstream land use, weather, hydrology, and water physicochemical properties, including fungicide levels. The dominant fungal communities and key environmental factors driving their dynamics were identified and discussed.

## 2 Materials and methods

### 2.1 Experimental design, sampling strategy, and metadata collection

**Figure 1:**
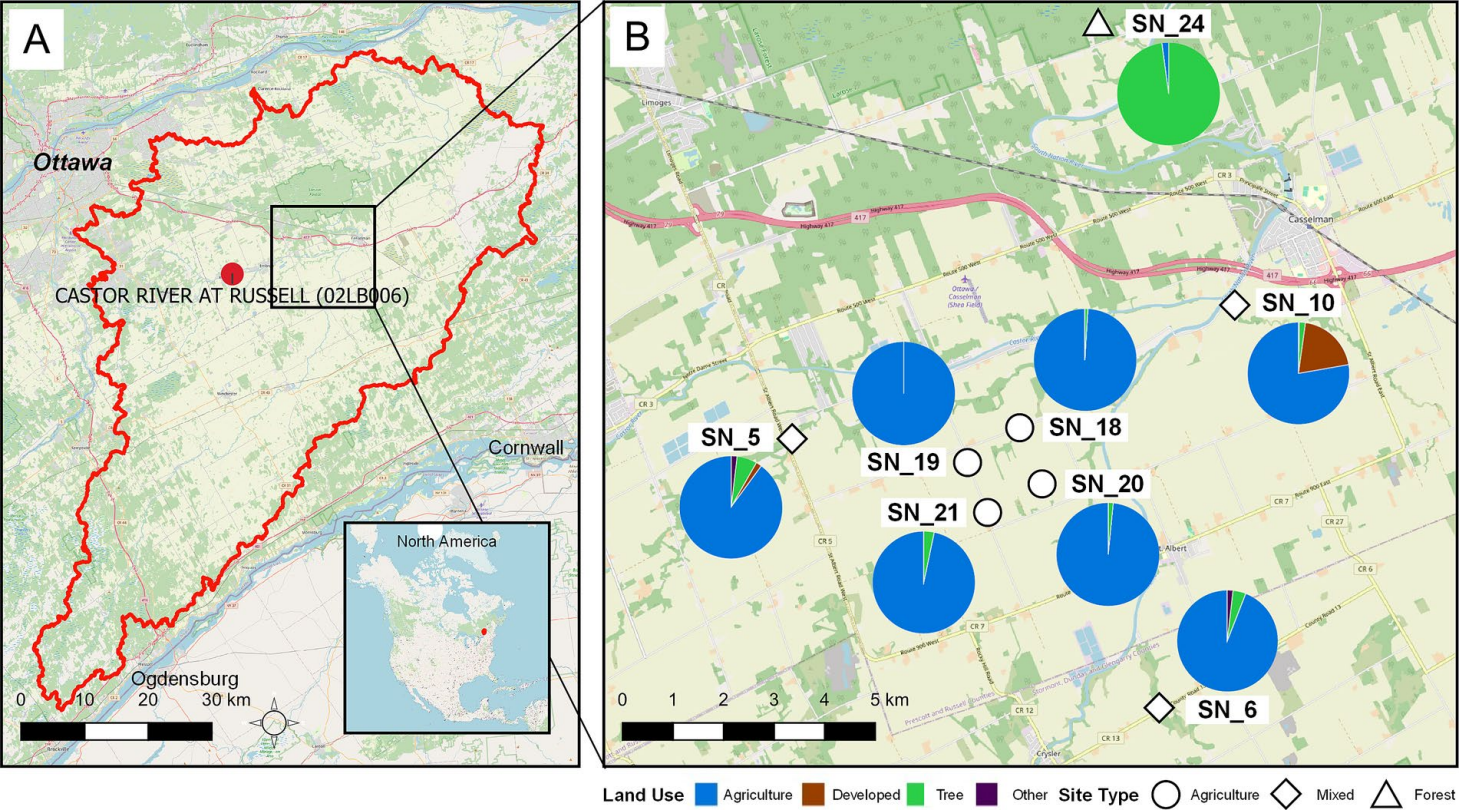
Geographic locations of sampling sites within the SNR basin with land use characterizations extending up to 5 km upstream from each site. (A) Map of the SNR basin, with the study area highlighted, and location of Russell station (02LB006), where Castor River discharge was measured, situated in eastern Ottawa, Ontario, Canada. (B) Sampling sites SN_18, 19, 20, and 21 are agricultural drainage ditch sites surrounded by agricultural fields, where SN_18/SN_19 and SN_20/SN_21 are, respectively, located within the same watershed. SN_5, 6, and 10 are influenced by mixed land uses and are situated at larger tributaries with nearby urban and agricultural lands and areas with tree cover. SN_24 is a small, forested stream characterized by minimal anthropogenic land use upstream. Map data copyrighted OpenStreetMap contributors and available from https://www.openstreetmap.org.

This study was conducted in the South Nation River (SNR) basin in Eastern Ontario, Canada (Figure 1). The SNR basin is approximately 3,900 km^2^ in size, and is characterized by agricultural land use, accounting for over 60% of the total land area, with small urban establishments and forest patches scattered throughout the region. Eight water sampling sites were selected to capture a gradient of land use features, stream order, and functions, as based on previous studies in the same water basin (Lapen et al., 2016; Chen et al., 2018; Shi et al., 2023).

The four agricultural water sampling sites (SN_18, SN_19, SN_20, and SN_21) are located on artificially constructed agricultural drainage ditches that receive almost exclusively tile drainage from adjacent arable fields (Sunohara et al., 2015; Sunohara et al., 2016). Sites SN_18 and SN_20 were sampled from 2016 to 2021, while sites SN_19 and SN_21 were sampled from 2017 to 2021. The mixed-land use water sampling sites (SN_5, SN_6, and SN_10) are located on larger order streams, with a mix of upstream land uses, including urban development, forested land, and agricultural land, relative to sites SN_18 through SN_21. The forested stream site (SN_24) receives water from a forested wetland with an upstream catchment area of <5 km^2^ and represents a site with no known agricultural or anthropogenic land uses (Lapen et al., 2016). This sampling site can be viewed as a reference or control site relative to the other water sampling sites as per Edge et al. (2012). Site SN_24 was the only forested water sampling site included in this study due to difficulties in accessing similar sites in this region and the requirement to process samples within 24 h of collection. The mixed land use (SN_5, SN_6, and SN_10) and the forested site (SN_24) are located on different sub-watersheds of the SNR basin and can be considered independent sampling sites, free from autocorrelation effects. However, the agricultural sites are distributed along two agricultural drainage ditches. SN_18 is located ~1.45 km downstream of SN_19, and SN_20 is located ~1 km downstream of SN_21, forming two pairs of pseudoreplicates. Therefore, SN_18 + SN_19 and SN_20 + SN_21 were considered paired sampling blocks, while all other sites were considered independent sampling blocks (*n* = 6, SN_5, SN_6, SN_10, SN_18 + SN_19, SN_20 + SN_21, SN_24). This approach was used in Shi et al. (2023) and Guo et al. (2024) for these sampling sites.

Surface water samples were collected biweekly between April and November over 6 years (2016–2021) (Supplementary Table S1). Samples were collected from 0 to 50 cm below the surface and transported on ice to the Ottawa Research and Development Centre of Agriculture and Agri-Food Canada (AAFC) for processing within 24 h. No water samples were collected when the ditches were dry or contained only disconnected puddles, typically in summer. The methods for water physicochemical property analysis and land use characterization have been detailed in Wilkes et al. (2011), with metadata summarized in Supplementary Table S2. Briefly, water temperature, pH, dissolved oxygen, conductivity, and turbidity were measured using a YSI 6600 multi-parameter water quality sonde at the time of collection (YSI Inc., Yellow Springs, OH). Portions of samples were sent to the Robert O. Pickard Environmental Centre (Ottawa, ON) for ammonia, ammonium, dissolved reactive phosphorus, total phosphorus, nitrite, nitrate, and total Kjeldahl nitrogen analysis. Analysis of total and dissolved organic carbon was performed based on the APHA 2540D method described by Rice et al. (2012). Stream order and land use were characterized by the percent catchment area for each sampling site, with distance of 2, 5, and 10 km upstream (Supplementary Table S3), using a Geographic Information System described in Wilkes et al. (2011) and Lyautey et al. (2010). Meteorological data, including air temperature, precipitation, and solar radiation, were collected at a weather station equipped with a HOBO data logger (Onset Computer Corp., Bourne, MS) near SN_20. Average temperature and cumulative precipitation were retrieved for the day of sampling as well as over 2, 3, 5, and 7 days prior to the sampling day. River discharge was recorded for the Castor River, running through the Russell station (Station 02LB006, 45°15′45″ N, 75°20′37″ W). This data was obtained from the Water Survey of Canada online archives (WSC, 2023) and served as a water flow proxy for the sample sites.

### 2.2 DNA extraction, library preparation, and Illumina MiSeq sequencing

To extract DNA from the water samples, 500 mL of each water sample was passed through a two-filter system to minimize clogging. First, water samples were filtered through a 0.7 μm borosilicate glass filter (Thermo Fisher, Ottawa, ON), where most fungal spores were captured. The filtrate was then passed through a 0.22 μm sterile nitrocellulose filter (Millipore, Billerica, MA, United States). DNA was extracted from each filter using DNeasy PowerSoil^®^ DNA extraction kits (Qiagen; formerly MoBio) following the manufacturer’s protocol. The extracted DNA was quantified using a Qubit 3.0 fluorometer, and purity was assessed by gel electrophoresis using 1% agarose gel with 1x TAE buffer (0.04 M Tris-acetate, 0.001 M EDTA, pH 7.8). DNA samples were stored at −80°C until further use.

Amplification of the internal transcribed spacer 2 (ITS2) region was performed by polymerase chain reaction (PCR) with the universal primer pair ITS9F (5′-GAA CGC AGC RAA IIG YGA-3′) and ITS4R (5′-TCC TCC GCT TAT TGA TAT GC-3′) (White et al., 1990; Menkis et al., 2012), and Qiagen HotStar MasterMix (Toronto, ON). PCR reactions included an initial denaturation step at 95°C, followed by 30 cycles of amplification (30-s denaturation at 95°C, 30-s annealing at 45°C, 30-s extension at 72°C), and a final 5-min extension at 72°C. PCR products were purified using NucleoMag NGS Clean-up and Size Select beads (Takara Bio Inc., Japan). The libraries were pooled in equimolar ratios, and diluted pools were prepared for sequencing following the manufacturer’s MiSeq System Denature and Dilute Libraries Guide. Sequencing was performed on the Illumina MiSeq system using a 500-cycle MiSeq Reagent Kit v2 at the National Research Council-Plant Biotechnology Institute (NRC-PBI, Saskatoon, SK), generating paired-end sequence reads of 250 bp in length.

### 2.3 Metabarcoding data processing

The raw sequencing data was processed using Quantitative Insights Into Microbial Ecology 2 (QIIME2, ver. 2021.11) (Bolyen et al., 2019). The quality of the raw sequencing reads was assessed using the q2-demux plugin to identify base positions where median sequencing quality exceeded a Phred score threshold of 35, indicating a base call accuracy above 99.96%. To meet this quality threshold, sequences were trimmed at 20 nt and truncated at 240 nt. Denoising was performed using the DADA2 denoise-paired QIIME2 plugin (Callahan et al., 2016) to merge paired reads and infer amplicon sequence variants (ASVs) using an expected error threshold of 2.0 and a chimera-fold abundance threshold of 2.0.

The representative sequences of the ASVs were further clustered into Operational Taxonomic Unit (OTUs) at a 97% similarity threshold using CD-HIT-EST (ver. 4.8.1) (Fu et al., 2012) to represent putative fungal species. This clustering was necessary because ASVs can overestimate fungal species diversity, as intraspecific variation within the ITS region may result in multiple ASVs representing a single species (Estensmo et al., 2021; Tedersoo et al., 2022). The OTU representative sequences were then processed using the fungal ITS extractor (ver. 2010.11) (Nilsson et al., 2010) to extract the ITS2 sequences and eliminate non-target amplifications. Remaining chimeras were removed using *uchime_ref* in UCHIME2 (ver. 4.2) (Edgar, 2016a) with a high confidence configuration, comparing with the UNITE fungal ITS database (ver. 9.0) (Nilsson et al., 2019). Taxonomic assignments for the OTU representative sequences were made using the Ribosomal Database Project (RDP) Naïve Bayesian classifier, implemented as the *classify. Seqs* command in mothur (ver. 1.48) (Wang et al., 2007; Schloss et al., 2009), with a minimum bootstrap confidence of 80% against the UNITE fungal ITS database version 9.0 (Nilsson et al., 2019).

OTU table curation and normalization followed these steps. First, OTUs from the 0.22 μm and 0.7 μm filters, along with any re-sequenced samples, were combined by summing the OTU read counts. The OTU table was then filtered to remove OTUs with total read counts below 10, eliminating potential chimeras or errors from PCR or sequencing (Edgar, 2016b; Tedersoo et al., 2022). OTUs unclassified to a known fungal phylum were also removed. Samples with fewer than 1,000 total reads were excluded, and total sum scaling was applied for community comparisons (McKnight et al., 2019). Rarefaction was not performed to avoid the unnecessary exclusion of rare taxa (McMurdie and Holmes, 2014).

### 2.4 Identification of known freshwater fungi and guild annotation of recovered fungi

To identify known freshwater fungi in the water samples, the names and habitat information of the aquatic fungal genera and species were compiled from Freshwaterfungi.org (https://freshwaterfungi.org/outline.php) and the Freshwater Ascomycetes database (https://fungi.life.illinois.edu/species_monographs) using a custom Python-based HTML text parsing script. Taxonomic lineages for fungal genera were retrieved from the National Center for Biotechnology Information (NCBI) taxonomy database using the R package myTAI (ver. 0.9.3) (Drost et al., 2018). A fungal genus missing NCBI taxonomy was manually cross-referenced with the Index Fungorum database (http://www.indexfungorum.org). Fungal genera without known taxonomic lineage information were excluded and not reported in the current study. OTUs’ taxonomic assignments were made at the genus level based on known aquatic fungi.

The FUNGuild database, accessed via the FUNGuildR package (ver. 0.2.0.9000) (Nguyen et al., 2016; Furneaux, 2021), was employed to assign OTUs to functional guilds. All annotations with confidence rankings of “Possible,” “Probable,” and “Highly probable” were retained. Fungal taxa were aggregated into “Saprotroph,” “Animal Pathogen,” and “Plant Pathogen,” representing the greatest relative abundance in this study.

### 2.5 Fungicides analysis

For the analysis of fungicides, 1.3 mL of surface water samples were transferred into a 2 mL amber HPLC vial and spiked with isotopically labeled internal standards to correct for sample-to-sample variation in signal suppression or enhancement (SSE%). Surface water samples were analyzed using online solid-phase extraction (SPE) with a Thermo Scientific Vanquish DUO HPLC coupled to an Altis triple quadrupole mass spectrometer. Each sample (950 μL) was injected onto a 2 cm Thermo Aq online SPE column. The trapped analytes were eluted from the online-SPE column onto an analytical Agilent Zorbax Eclipse Plus HPLC column (2.1 × 50 mm, 1.8 μm) at 35°C with a flow rate of 300 μL min^−1^. A gradient mobile phase was used, starting with mobile phase A (H_2_O + 0.1% FA; Optima LC–MS Grade) held at 99% for 0.750 min, followed by an increase of mobile phase B (acetonitrile +0.1% FA; Optima LC–MS Grade) to 15% over 1.05 min, 24% over 5.6 min, and 98% over 15.1 min, held for 2.4 min. The OptaMax NG HESI source was operated with capillary voltages of 3.5 kV in both positive and negative mode, an ion transfer tube temperature of 325°C, and a vaporizer temperature of 350°C. Sheath, auxiliary, and sweep gasses were set to 35, 10, and 1 arbitrary units, respectively. Target analytes and corresponding internal standards were monitored as detailed in Supplementary Table S4. Quantification was performed in Thermo TraceFinder 5.0.

### 2.6 Statistical analysis

All statistical analyses, unless stated otherwise, were performed in R (ver. 4.2.1) (R Core Team, 2022). Missing water physicochemical data were imputed using random forest imputation with the missForest package (ver. 1.5) (Stekhoven and Buhlmann, 2012). *p* values were adjusted for multiple comparisons using the Benjamini-Hochberg method (Benjamini and Hochberg, 1995), with *p* ≤ 0.05 being interpreted as statistically significant.

To visualize the discrimination of sampling sites by water physicochemical properties, we applied Partial Least Squares Discriminant Analysis (PLS-DA) using the DiscriMiner package (ver. 0.1–29) (Sanchez, 2013). OTU diversity and sample representation were assessed using the iNEXT package (ver. 2.0.20) to generate rarefaction curves (Hsieh et al., 2016). Alpha- and beta- diversity indices were calculated using the vegan package (ver. 2.6–2) (Oksanen et al., 2020). The Shannon-Wiener (SW) diversity index was transformed into true diversity (TD) using the following formula: SW-TD = exp.(SW), as described by Jost (2006). SW-TD was used as a measure of alpha diversity, representing within-sample species richness (Whittaker, 1972). Environmental drivers of alpha diversity were identified using random forest models with the randomForest package (ver. 4.7-1.1) (Liaw and Wiener, 2002). Community ordination relative to water physicochemical and meteorological properties was conducted using distance-based redundancy analysis (dbRDA) in the vegan package, with Aitchison distance used to capturing dissimilarities between samples. OTU abundance tables were centered log-ratio (clr)-transformed to manage compositional biases (Aitchison, 1982). Environmental data were normalized to a mean of 0 and variance of 1 using the *decostand* function in vegan with the “standardize” method prior to multivariate analysis.

The significance of land use classes on community structure was assessed using permutational multivariate analysis of variance (perMANOVA) with function *adonis2* in vegan, excluding variables with variance inflation factors greater than 10. Partial redundancy analysis (partial RDA) was performed to separately model the community variance explained by water physicochemical properties and fungicide concentrations. Each group of variables was treated as a constraint in individual partial RDA models, while the other variables were controlled as covariates. The significance of the variance explained by each group of variables was then tested using ANOVA on the partial RDA models, allowing for the assessment of each group’s contribution to community variance. Importance of environmental factors driving the beta-diversity were analyzed using the gradientForest package (ver. 0.1-34) (Ellis et al., 2012). Temporal changes in community composition were analyzed using the *multivariate_change* function from the codyn package (ver. 2.0.5) (Hallett et al., 2016). Spearman’s rank correlation (*ρ*) was used to assess variable correlations. Differences in alpha diversity, taxon abundance (clr-transformed), and water physicochemical properties across land use classes were analyzed using linear mixed-effects (LME) models with the *lme* function in the nlme package (ver. 3.1-162) (Pinheiro et al., 2023). Prior to running the LME model, we conducted normality tests on these parameters and data were transformed as needed using the *boxcox* function from the MASS package (ver. 7.3-60.4) (Ripley et al., 2013). These LME models used land use classes as fixed effects and sampling date and sample blocks as random effects. The normality of the residues from the LME models was tested to ensure the model assumptions were met. ANOVA was used to test the significance of factors in LME models, with post-hoc pairwise comparisons between land use classes performed using the *emmeans* function from the emmeans package (ver. 1.8.4-1) and the “Tukey” method for *p-*value adjustment (Lenth, 2023). The Tukey test was also used for all pairwise comparisons via the *TukeyHSD* function (R Core Team, 2022). Seasonal dynamics in alpha diversity and taxon abundance were visualized using *stat_smooth* in ggplot2 (ver. 3.4.1), applying “loess” smoothing for their trends (Wickham, 2016).

For exploratory analysis, classification and regression tree (CART) analysis (Salford Systems, San Diego, CA, United States) was used to investigate fungicide associations with Shannon and Simpson diversity indices and fungal genera abundances. CART, a non-parametric method, partitions data into nodes (groups) in a binary and recursive manner based on independent variables (Lapen et al., 2001; Wilkes et al., 2013).

## 3 Results

### 3.1 Impact of land uses on water physicochemical properties

Given the importance of water physicochemical properties in shaping environmental microbiomes, we first assessed the stream water quality across the different sites. Water properties at the forested site (SN_24) differed significantly from those at the mixed-use and agricultural ditch sites, which had similar characteristics due to the influence of, primarily, agricultural activities (Supplementary Figures S1–S3; Table 1; Supplementary Table S3). Specifically, total and dissolved organic carbon concentrations were significantly higher at the forested site, likely influenced by wetland effects, whereas water conductivity was lower compared to the agricultural and mixed-use sites (*p* < 0.05) (Supplementary Figure S1; Table 1). Nitrate concentrations peaked during the spring and fall at the agricultural and mixed-use sites, correlating with fertilizer applications and drainage or runoff events (Supplementary Figure S1; Table 1).

**Table 1:** Comparison of water physicochemical properties by land use class across all sampling years using linear mixed effects model.

Water property	ANOVA *p* value	Pairwise comparison between land use classes		
		Agr-mixed *p* value	Agr-forest *p* value	Mixed-forest *p* value
AMIA_AMN	0.1145	0.0681	0.1311	0.9799
CONDUCTIVITY_MSC	0.0815	0.2012	0.0168*	0.0344*
DISS_OXYGEN_MGL	0.8751	0.9994	0.8753	0.8839
DOC	0.0370*	0.5970	0.0059*	0.0065*
NITRATE	0.0919	0.4269	0.0589	0.0256*
NITRITE	0.5373	0.5920	0.4745	0.8467
ORP_MV	0.6984	0.6953	0.9988	0.7503
PH	0.5021	0.3631	0.6960	0.9273
TEMP_C	0.0919	0.0821	0.3979	0.0417*
TOC	0.0370*	0.5889	0.0060*	0.0066*
TOTKN	0.1584	0.1041	0.1891	0.9919
TOTPHO	0.4839	0.4543	0.3562	0.8014
TURBIDITY_NTU	0.0957	0.0517	0.0860	0.8996

ANOVA was performed on a linear mixed effects model where land use class was a categorical fixed effect; sampling date and sampling block were random effects. Pairwise comparisons were performed by estimated marginal means with Tukey *p* value adjustment. Significance levels are indicated in bold text, and are denoted as *p ≤0.05. Variable descriptions for water physicochemical properties are listed in Supplementary Table S2.

River discharge at the Castor River was used as flow proxy for the ungauged sample sites (Wilkes et al., 2011). The year 2016 was unusually dry, with sparse rainfall events compared to other sampling years, as evidenced by cumulative precipitation and river discharge data (Supplementary Figure S4A). These dry conditions limited sample acquisition at the shallow agricultural drainage ditch sites. In contrast, 2017 was a wet year with frequent heavy rainfall events (>600 mm cumulative), allowing for more samples to be collected from agricultural drainage ditch sites (Supplementary Table S1).

A total of 11 fungicides, each at concentrations below 1 μg/L, were detected at agricultural and mixed-use sites between 2018 and 2020, with the majority collected in 2019. Boscalid and Metalaxyl were the most frequently detected at the highest concentrations. None of the detected fungicides differed significantly in concentration between the agricultural and the mixed-use sites (Table 2). Fungicide analysis was not performed on samples from the forested site (SN_24).

**Table 2:** Descriptive statistics of prevalent fungicide compounds at agricultural ditch and mixed-use sites.

Compound	Agriculture		Mixed		*p*-value^a^
	Mean (ng L^−1^)	SD (ng L^−1^)	Mean (ng L^−1^)	SD (ng L^−1^)	
Azoxystrobin	0.55	2.98	0.17	0.42	0.8281
Boscalid	34.02	132.73	1.23	3.68	0.8281
Difenoconazole	0.96	1.15	1.55	2.00	0.8281
Metalaxyl	12.91	35.02	9.43	9.68	0.8281
Picoxystrobin	0.31	1.19	0.40	1.27	0.9682
Propiconazole	1.03	4.74	0.51	0.97	0.8281
Pyraclostrobin	6.66	6.44	8.58	5.09	0.8281
Spiroxamine	0.58	1.01	0.34	0.63	0.8281
Tebuconazole	4.51	7.04	3.22	7.87	0.8281
Thiabendazole	0.04	0.21	0.06	0.19	0.8281
Trifloxystrobin	1.96	7.23	2.20	1.81	0.9682

a *p-value represent the effect of land use class on difference in mean fungicide concentration using linear mixed effect modeling.*

### 3.2 Freshwater mycobiota

The stream mycobiota recovered through metabarcoding included both true aquatic fungi and terrestrial fungi. The final OTU table contained 6,571 OTUs, representing 10,021,213 high-quality reads, from 503 samples, with each sample averaging 19,922 ± 20,737 (MEAN ± SD) reads. The cumulative rarefaction curve suggested nearly complete recovery of OTUs for each sampling site, indicating additional sampling would not improve OTU recovery (Supplementary Figure S5). Majority of the OTUs were assigned to three phyla: Ascomycota (87% of total reads, 74% of OTUs), Basidiomycota (9, 19%), and Chytridiomycota (4, 5%). The remaining fungal phyla accounted for less than 1% of the reads and 2% of the OTUs (Supplementary Figure S6). Ascomycota were more abundant at agricultural and mixed-use sites, whereas Basidiomycota were more common at the forested site (Supplementary Figure S6). Approximately one-third of OTUs (1,720) were found across all land use classes, accounting for 26% of total abundance (Supplementary Figure S7). The most abundant genera recovered (≥2.5%) include *Cladosporium* (4.25 ± 6.47%), *Ramularia* (4.02 ± 9.40%), *Alternaria* (2.82 ± 6.19%), and *Sarocladium* (2.71 ± 12.7%) (Supplementary Figure S8).

As of July 15, 2023, the Freshwaterfungi and the Freshwater Ascomycetes databases contained 284 genera containing true aquatic fungal species. In the current study, 138 OTUs were assigned to 58 out of the 284 fungal genera, represented a small fraction of known aquatic fungal lineages. These OTUs, accounting for ~3% of total abundance, primarily belonged to the Dothideomycetes and Sordariomycetes classes. *Phaeosphaeria*, *Biappendiculispora*, and *Trichoderma* were recovered across all sample locations, with insignificant differences in abundance (*p* > 0.05, Table 3). Notably, *Phaeosphaeria* was one of the most abundant fungal genera recovered in this study.

**Table 3:** Habitat descriptions of most abundant fungal genera that contain aquatic fungal species.

Genus	# of OTUs	Total relative abundance (%)	Mean relative abundance (%)^a^			*p* value^b^	Trophic mode	Fungal guild	Habitat description
			Agr	Mixed	Forest				
*Phaeosphaeria*	12	2.4055	2.6633	1.1552	0.7691	0.0509	Pathotroph-Saprotroph	Fungal Parasite-Plant Pathogen-Plant Saprotroph	Diverse habitats in freshwater environments
*Biappendiculispora*	2	0.1646	0.3882	0.0892	0.1530	0.2774	Pathotroph-Saprotroph	Plant Pathogen-Undefined Saprotroph	Submerged wood or dead herbaceous grass stems
*Trichoderma*	9	0.1421	0.0947	0.0117	0.1768	0.3813	Pathotroph-Saprotroph-Symbiotroph	Animal Pathogen-Endophyte-Epiphyte-Fungal Parasite-Plant Pathogen-Wood Saprotroph	Diverse habitats, including soil, wood, freshwater and marine water
*Myrmecridium*	7	0.1242	0.1915	0.0711	0.0483	0.0718	Saprotroph	Undefined Saprotroph	Submerged decaying wood, leaf litter, stems, and leaves of herbaceous plants
*Nigrograna*	3	0.0853	0.0661	0.1822	0.0152	0.4058	Pathotroph	Animal Pathogen	Submerged wood from twigs of shrubs and trees
*Crassiclypeus*	2	0.0759	0.1464	0.0891	0.0200	0.6879	Pathotroph-Saprotroph	Plant Pathogen-Undefined Saprotroph	Submerged dead twigs of woody plants
*Periconia*	7	0.0749	*NA* ^c^	*NA* ^c^	*NA* ^c^	*NA* ^c^	Pathotroph-Saprotroph-Symbiotroph	Endophyte-Plant Pathogen-Wood Saprotroph	Decaying wood in terrestrial, freshwater, mangrove, and marine environments
*Trematosphaeria*	2	0.0448	0.0091	0.1454	0.0006	0.6304	Saprotroph	Undefined Saprotroph	Terrestrial or submerged wood in freshwater
*Nectria*	1	0.0409	0.1427	0.0305	0.0146	0.5021	Pathotroph-Saprotroph-Symbiotroph	Animal Pathogen-Endophyte-Fungal Parasite-Lichen Parasite-Plant Pathogen-Wood Saprotroph	Diverse habitats, including wood bark, dead wood, submerged twigs
*Lentithecium*	1	0.0320	0.0071	0.0586	0.0491	0.2102	Saprotroph	Wood Saprotroph-Plant Saprotroph	Submerged wood, grass in freshwater or terrestrial environments

a Mean relative abundance computed by estimated marginal mean from linear mixed effects model.

b *p values represent the effect of land use class on difference in mean relative abundance using linear mixed effect modeling.*

c NA values were introduced due to insufficient data to run linear mixed effects model.

### 3.3 Impact of land uses on freshwater mycobiota diversity and function

**Figure 2:**
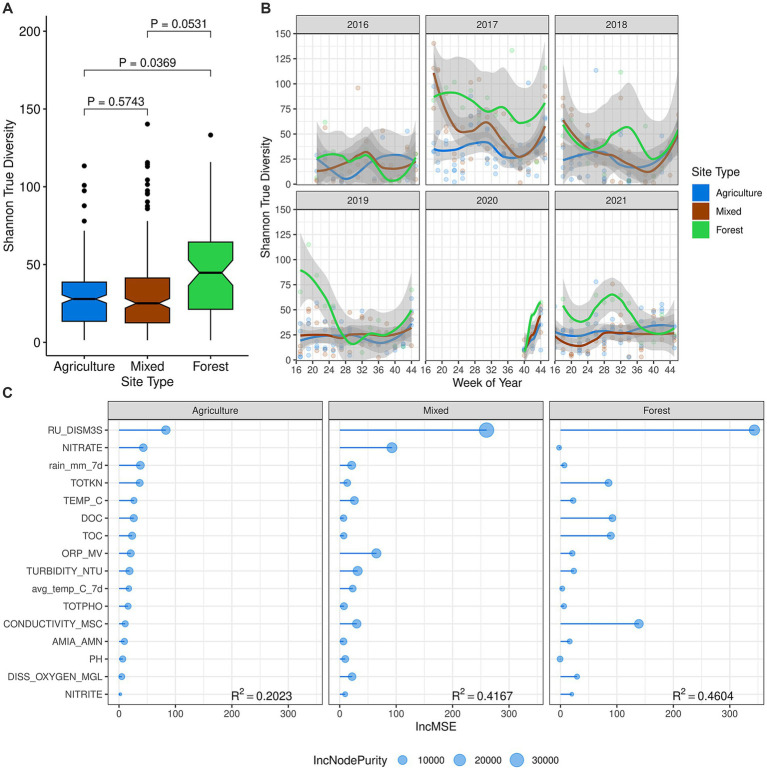
Alpha diversity of surface water samples. (A) Mean alpha diversity, measured using the Shannon-Wiener True Diversity (SW-TD) value. Differences in means between agricultural, mixed-use, and forested land use classes were compared using linear mixed-effects models. (B) The seasonal dynamic trend of SW-TD fitted using local polynomial regression across all sampling years. (C) The importance of environmental variables and their contribution to alpha diversity evaluated using random forest regression, computed for each land use class. The order of environmental variables was determined based on their importance at agricultural drainage ditch sites, measured as the Increase in Mean Squared Error (IncMSE). The *R*^2^ value of the final Random Forest model is reported for each land use class. Variable descriptions for water physicochemical properties are listed in Supplementary Table S2.

Building on our previous findings that higher bacterial diversity was associated with subsurface drinking water intake and water recreation sites compared to agricultural ditch sites (Chen et al., 2018), we observed similar patterns in stream fungal communities. Alpha diversity, assessed using the Shannon-Wiener True Diversity (SW-TD) value, was highest at the forested site, moderately high at mixed-use sites, and lowest at agricultural drainage ditch sites (forest-mixed, *p* = 0.0531; forest-agr, *p* = 0.0369) (Figure 2A). Fungal diversity varied notably among sampling years, with the lowest diversity observed in 2016, particularly at the forested site (Figure 2B). Alpha diversity often peaked at the onset of the sampling season or during the warmer summer periods, likely linked to water flow events (Supplementary Figure S4B).

Random Forest models revealed greater explanatory power of environmental factors at mixed-use (*R*^2^ = 0.42) and forested (*R*^2^ = 0.46) sites compared to agricultural drainage ditch sites (*R*^2^ = 0.2023) (Figure 2C), suggesting unexplored factors may have influenced fungal diversity at the latter. River discharge emerged as the primary driver of fungal diversity, positively correlated with alpha diversity across all land use classes (agricultural ditches, *ρ* = 0.36, *p* < 0.0001; mixed-use, *ρ* = 0.52, *p* < 0.0001; forested, *ρ* = 0.67, *p* < 0.0001) (Figure 2C; Supplementary Figure S9). Water nitrate concentration positively correlated (*p* ≤ 0.05) with river discharge at agricultural drainage ditch sites and mixed-use sites, but not at the forested site, where anthropogenic influence was minimal. Conversely, conductivity, total and dissolved organic carbon, and total nitrogen concentration promoted the stream fungal diversity at the forested site (Supplementary Figure S9).

**Figure 3:**
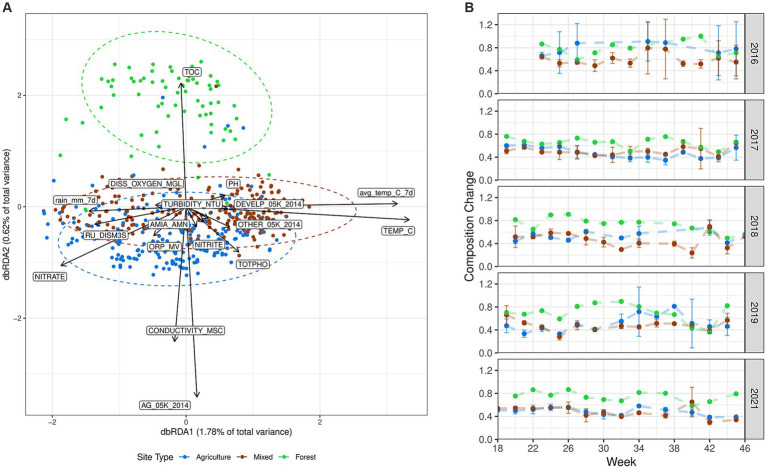
Changes in stream fungal community compositional structure. (A) dbRDA of the surface water fungal community in the SNR basin based on Aitchison distance of centered log-ratio (clr) transformed OTU table and standardized environmental metadata, showing the contribution of dbRDA axes to the total community variation. The overall adjusted *R^2^* of the dbRDA model was 0.025. (B) Changes in community compositional structure between sampling time points, expressed as Aitchison distance between centroids of replicates. Error bars represent the dispersion change or average distance between replicates and their centroids at each time point. No error bars are reported for forested sites due to *n* = 1 replicate per time point. The year 2020 was excluded from the analysis of compositional structure changes due to under-sampling during the COVID-19 lockdowns. Variable descriptions for water physicochemical properties are listed in Supplementary Table S2.

The stream mycobiota composition differed significantly across land use classes, as shown by perMANOVA results (ADONIS2, *F* = 16.15, *p* = 0.037). The dbRDA analysis clearly separated samples from the forested site from those of the agricultural drainage ditch sites and mixed-use sites (Figure 3A). Gradient Random Forest analysis identified river discharge and water temperature as the most important factors influencing fungal community compositional structure. However, these environmental factors together explained only a small portion of the community variation (*R*^2^ < 0.02 for gradient forest and *R*^2^ = 0.025 for dbRDA), suggesting that unmeasured factors may play critical roles in shaping the stream mycobiota in the SNR basin (Supplementary Figure S10).

**Figure 4:**
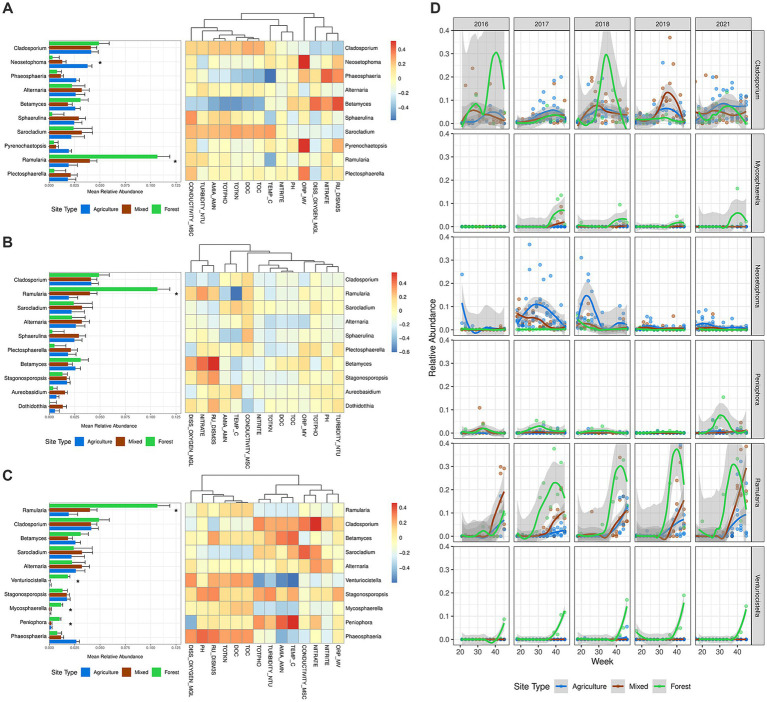
Relative abundance and seasonal dynamics of the most abundant genera with correlations to water physicochemical properties between land use classes. (A–C) Relative abundance of the top 10 most abundant genera with correlation heatmap of relative abundance with water physicochemical properties at (A) agriculture sites, (B) mixed-use sites, and (C) forested site. The mean relative abundance was computed with estimated marginal mean of the linear mixed effect models, where whiskers denote standard error. Differences in mean relative abundance were computed with linear mixed effects models, where significant difference in mean relative abundance due to the effect of land use class are denoted *p < 0.05. The columns of the correlation heatmap were clustered using the “hclust” method for each land use class. (D) Temporal variation in selected genera over time by land use class with a fitted local polynomial regression line. Fungal genera were selected due to a significant difference in mean relative abundance, except for *Cladosporium*, which was selected due to having the highest mean relative abundance. Year 2020 was not plotted for analysis of temporal variations due to under sampling during the COVID-19 lockdowns. Variable descriptions for water physicochemical properties are listed in Supplementary Table S2.

Among the most abundant fungal genera, *Ramularia* was notable. It was most abundant at the forested site (averaging 10.67% in abundance) and significantly less so at mixed-use (4.01%, *p* = 0.0357) and agricultural drainage ditch sites (1.91%, *p* = 0.0204) (Figures 4A–C; Supplementary Figure S8). *Ramularia* abundance peaked in the summer (late July to early August) (Figure 4D; Supplementary Figure S4A). At agricultural drainage ditch and mixed-use sites, *Ramularia* abundance was negatively correlated with water temperature, and total Kjeldahl nitrogen concentration, but positively correlated with water conductivity and nitrate concentration. These trends, however, were not observed at the forested site (Figures 4A–C). *Venturiocistella*, *Mycosphaerella*, and *Peniophora* were also more abundant at the forested site, with *Venturiocistella* increasing in abundance starting in early October when temperature began to drop (*ρ* = −0.60, *p* < 0.0001). No clear seasonal patterns were observed for *Mycosphaerella* and *Peniophora*. In contrast, *Neosetophoma* was found more abundant in agricultural drainage ditches (3.79% in abundance) compared to mixed-use sites (1.29%, *p* = 0.0445) and the forested site (0.33%, *p* = 0.0437) (Supplementary Figure S8).

**Figure 5:**
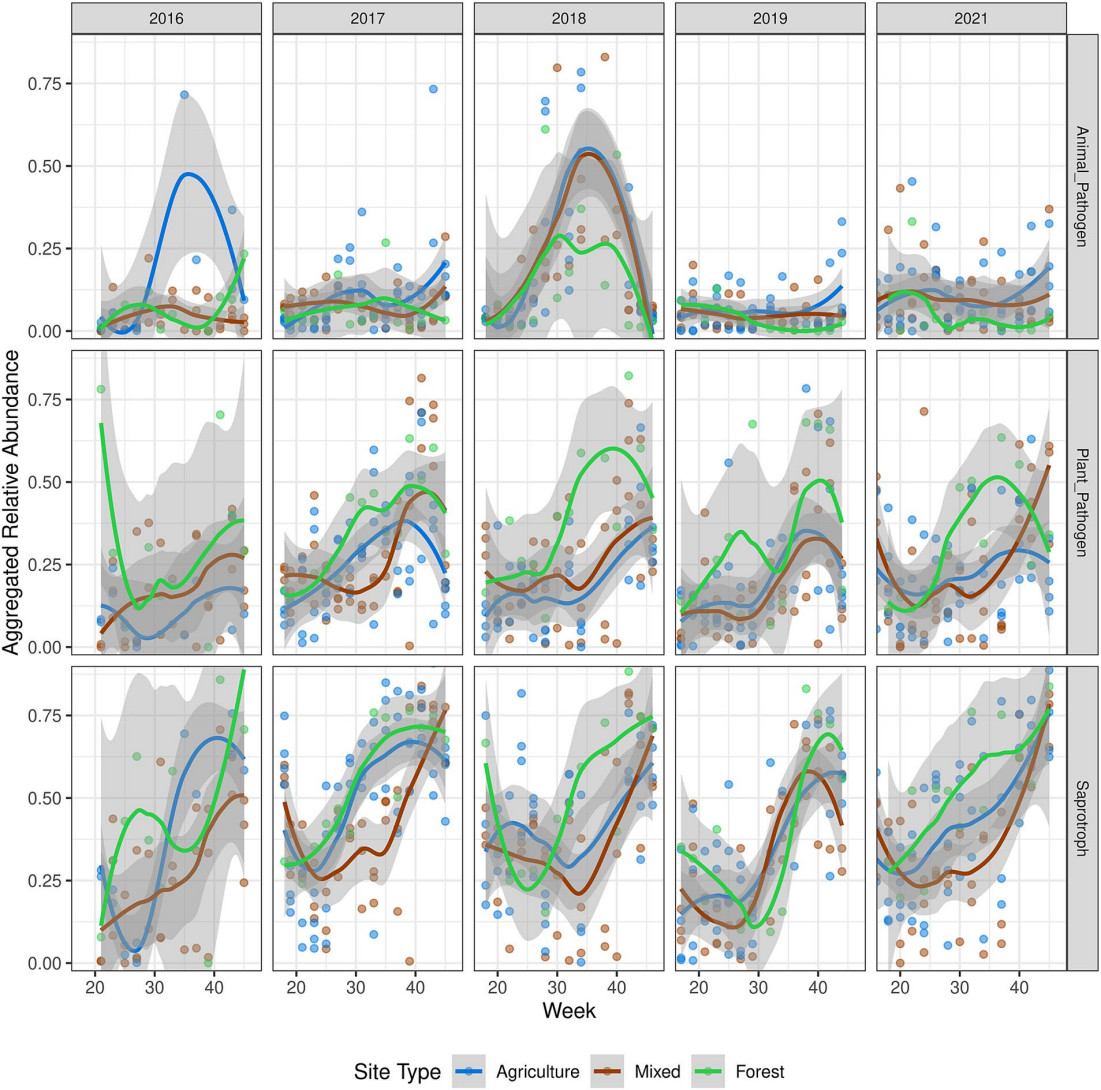
Dynamics of functional guild abundance. OTUs annotated using FUNGuild with guilds containing “animal pathogen,” “plant pathogen,” or “saprotroph,” were aggregated together, resulting in an aggregated relative abundance measure. Trend lines were fitted using loess regression, and the shaded area represents the standard error. The year 2020 was not plotted due to under-sampling during the COVID-19 lockdowns.

Next, we annotated the ecological functionality of 2,498 OTUs, representing 801 fungal genera, using the FUNGuild database (Nguyen et al., 2016). The most abundant guilds were saprotrophs, animal pathogens, and plant pathogens (Supplementary Figure S11), with no significant association with land use classes, indicating potential functional redundancy. Seasonal trends were observed in the abundance of saprotrophs and potential plant pathogens, such as *Cladosporium*, *Ramularia*, and *Alternaria*, which increased from late July or early August (around week 30) and peaked toward the end of autumn (Figure 5). In contrast, potential animal pathogens showed no clear seasonal pattern, except for a notable peak in summer 2018, primarily driven by an increase in *Sarocladium* spp. (Supplementary Figure S12).

### 3.4 Impact of fungicide levels on stream mycobiota

During the 2018–2020 sampling years, when fungicides were analyzed at agricultural drainage ditch and mixed-use sites (excluding the forested site), fungicides had a statistically significant impact on the stream fungal communities, as indicated by the partial RDA result (*F* = 1.2236, *p* = 0.003). However, fungicides explained only 2.5% of the variation in compositional structure, whereas water physicochemical properties accounted for 20%, with their interaction explaining an additional 2.5%.

CART analysis identified the most influential fungicides on fungal alpha-diversity. Difenoconazole concentration was the primary splitter of Simpson-True Diversity (Supplementary Table S6), showing a positive correlation. Higher fungicide concentrations might be associated with greater runoff and microbial inputs from adjacent fields, potentially increasing diversity, though this trend was not consistent across all fungicides. For example, Propoconazole was the primary splitter for Shannon Weiner True Diversity, where higher concentrations equated to lower diversity (Supplementary Table S6). Linear regressions between these diversity values and 7-day precipitation (a surrogate for potential overland flow and tile drainage) showed low explanatory power (*R*^2^ < 0.04), indicating that rainfall poorly explained the variation in diversity.

Additionally, CART identified the most important fungicides affecting the relative abundances of prevalent fungal genera. For *Ramularia* and *Betamyces*, Metalaxyl, and Difenoconazole were the primary splitters, respectively, where higher fungicide concentrations were associated with lower relative abundance. In contrast, for *Sphaerulina, Neosetophoma, Peniophora*, and *Venturiocistella*, the primary fungicide splitters were Tebuconazole, Metalaxyl, Picoxystrobin, and Pyracolstrobin, respectively, with higher fungicide concentrations linked to greater relative abundance (Supplementary Table S7). Linear regressions of fungal genera abundances and 7-day precipitation revealed low positive slopes for *Ramularia, Sphaerulina, Peniophora*, and *Venturiocistella,* and low negative slops for *Betamyces* and *Neosetophoma*. All regressions showed low *R*^2^ values (<0.1), indicating rainfall poorly explained the variation in fungal genera abundances.

## 4 Discussion

By integrating fungal metabarcoding data with information on land use, hydrology, weather, and water physicochemical attributes across streams within the agriculturally dominated SNR basin in Eastern Canada, we uncovered significant disparities in fungal diversity and community composition among agricultural drainage ditch, mixed-use, and forested/wetland-dominated systems. Notably, despite the recognized importance of these environmental factors, they accounted for only a small portion of the variation in stream fungal community composition. Our study suggests unmeasured factors and stochastic processes may play a significant role in shaping stream mycobiota. Additionally, we identified specific terrestrial fungal genera entering stream, which could serve as indicators of runoff, tile drainage, or soil erosion from adjacent lands.

### 4.1 Freshwater fungi in the South Nation River basin under agricultural impact

One of the research objectives of this study was to identify fungal genera containing known freshwater fungal species by referencing two databases: the Freshwater Ascomycetes database (Shearer and Raja, 2010) and freshwaterfungi.org (Calabon et al., 2020). However, the limited entries in these databases, compared to the broader known diversity of aquatic fungal species (Calabon et al., 2023), likely led to an underestimation of aquatic fungi in our dataset. Despite this, we identified 138 OTUs across 58 genera potentially containing aquatic fungal species at low abundance (3% of the total abundance). These genera, typically colonizing submerged wood or herbaceous stems, showed no significant differences in abundance across different land use classes (Table 3), suggesting they are widespread in the region (Calabon et al., 2020). This is expected, as all sites had abundant submerged organic materials, typical substrates for the observed genera. Some genera, such as *Phaeosphaeria* and *Nectria*, are diverse and include species that are not exclusively aquatic (Table 3).

Our findings indicate that the fungal communities in this study comprised both true aquatic and transient terrestrial fungi in freshwater environments. The majority of the detected fungal taxa were terrestrial, such as *Cladosporium* and *Ramularia*, likely due to the proximity of the surface water collection points to land, increasing the influx of terrestrial fungi into the streams. This may also explain why our study recovered chytrids in low abundance compared to studies that sampled lakes and larger streams (Bai et al., 2018; Lepere et al., 2019; Chen et al., 2020). Our lower detection of chytrids may be also due to primer bias and the choice of barcode regions. Previous studies, such as Bai et al. (2018) and Chen et al. (2020), targeted ITS1, while Lepere et al. (2019) used 18S rRNA gene region, whereas our study targeted the ITS2 region with the ITS9F/ITS4R primer pair. Chen W. et al. (2022) noted that the ITS4R primer perfectly matches only 45.2% of Chytridiomycota ITS2 sequences in the NCBI GenBank database, while Heeger et al. (2019) highlighted the challenges in effectively recovering fungi from this phylum using only the ITS2 barcode. It is also worth noting that ITS sequences of chytrids are underrepresented in the UNITE ITS reference database used in this study, compared to those of other fungal phyla.

### 4.2 Impact of dominant upstream land uses on fungal communities

Alpha diversity of stream fungi is an essential metric reflecting the community’s ecological functions, with higher diversity supporting a diverse array of decomposers and saprotrophs, which form stable biofilm communities and enhance resilience and stability in stream ecosystems (Besemer, 2015). Our study showed that stream fungal diversity was highest at the forested site (SN_24) compared to agricultural drainage ditch sites and mixed-use sites (Figure 2A). This is consistent with previous studies indicating that microbial diversity is highest in minimally disturbed environments, such as mountainous regions or national parks, compared to those impacted by anthropogenic activities like agriculture and urbanization (Bärlocher et al., 2010; Bai et al., 2018). Additionally, the stream fungal community composition significantly differed between the forested site and the agriculturally impacted sites (drainage ditches and mixed-use sites) (Figure 3A). Bai et al. (2018) and Ortiz-Vera et al. (2018) further demonstrated that aquatic fungal community structures in anthropogenically impacted freshwater ecosystems are distinct from those in relatively undisturbed environments.

In this study, Ascomycota was significantly more abundant at the agricultural ditch and mixed-use sites than the forested site, while Basidiomycota abundance showed an opposite trend (Supplementary Figure S6). Such differences may reflect substrate availability and nutrient composition at anthropogenically impacted sites and a greater capacity for the transport of particulates from source areas to water pathways. For example, agricultural ditch sites and mixed-use sites receive inputs of agrochemicals and crop residues, favoring the proliferation of Ascomycota, known for adapting to nutrient-rich environments (Solé et al., 2008). These sites, located in cropland areas, may experience more extreme windblown deposition of airborne particulates compared to more protected forested sites. In contrast, forested sites provide more woody debris and dissolved organic matter, promoting Basidiomycota decomposers (Goodell, 2020). This contrasts with Bai et al. (2018), who observed no significant difference in the relative abundance of Ascomycota and Basidiomycota when comparing communities in a less developed mountainous region and downstream areas with increased urban and agricultural land use in the Chaobai River basin, Beijing, China.

Noteworthy differences in dominant genera emerged between land use classes (i.e., agricultural ditch sites, mixed-use sites, vs. forested site), suggesting adaptation to site-specific environmental conditions. Fungal genera such as *Venturiocistella*, *Mycosphaerella*, and *Peniophora* were predominantly abundant in the forested site but were scarce in agricultural drainage ditch and mixed-use sites (Figures 4A–C). These saprotrophic fungi play crucial roles in nutrient recycling and organic matter decomposition. In particular, *Peniophora* spp. can colonize dead wood and are commonly affiliated with wood decay in forests and other natural habitats (Lambevska et al., 2013). Many species in *Mycosphaerella* and *Peniophora* are also plant pathogens, capable of infecting crops and forest trees (Cannon and Kirk, 2007; Crous et al., 2009; Nguyen et al., 2016). These genera were positively correlated with total organic carbon, which was elevated in the forested site due to the wetland present there. The abundances of *Mycosphaerella* and *Venturiocistella* were negatively correlated with ammonia, nitrate, and phosphate, which were observed in higher concentrations at agriculturally impacted sites (agricultural ditch sites and mixed-use sites), implying their sensitivity to these pollutants. This is corroborated by Mondal et al. (2007), who found that *Mycosphaerella* species were sensitive to nitrogen enrichment, which reduced their biomass.

*Ramularia* was the only genus found in high abundance across all sites but was notably more abundant in the forested site. In contrast, *Neosetophoma* and *Sphaerulina* were prominent in agricultural drainage ditch sites and mixed-use sites during certain sampling periods (*Neosetophoma*, weeks 20–30; *Sphaerulina* weeks 30–40; Supplementary Figures S8, S12), correlating positively with water conductivity, an indicator of water nutrient levels. Species of these two genera can be foliar pathogens with a broad host range (Cannon and Kirk, 2007). Their higher abundance in agricultural sites indicates a tolerance to environmental stressors like soil erosion and runoff of agrochemicals. However, despite access to a broader range of hosts, they may be outcompeted in forested sites by a more diverse fungal community, likely arises due to competitive exclusion (Hardin, 1960), leading to their lower abundance in these areas.

In summary, agricultural land use appears to be associated with lower diversity in stream fungal communities, likely due to shifts in the abundance of various taxa driven by anthropogenic effects on water quality and nutrients.

### 4.3 Environmental drivers of the freshwater fungal community

Several environmental factors significantly influenced the stream fungal communities. Water discharge, driven by precipitation events, was identified as the primary driver of both alpha- and beta-diversity (Figure 2C; Supplementary Figure S10). Our study found that total and dissolved organic carbon levels were more than twice as high at the forested site compared to agricultural drainage ditch sites and mixed-use sites. These dissolved organic carbon levels positively correlated with fungal diversity at the forested site (Figure 2C; Supplementary Figure S9). The diverse riparian and basin plants in forested and wetland areas contribute a wide variety of organic matter (Hagen et al., 2010), which can be transported into the stream via precipitation and aerial deposition (Aitkenhead-Peterson et al., 2003), leading to increased fungal diversity and biomass in streams (Fernandes et al., 2013; Danger et al., 2016). Additionally, dead plant material on soil surfaces can create habitats conducive to the growth of aquatic fungi, which are then carried into the stream by erosion events (Chauvet et al., 2016). Instances of heightened diversity corresponded with increased river discharge, serving as a surrogate for precipitation influences on transport, at the beginning of the sampling season, especially at the forested site during wetter spring conditions (Figure 2B; Supplementary Figure S4A). Similar diversity spikes occurred mid-season during rainier summer months, notably in 2017 (Supplementary Figure S4A). In contrast, the reduced diversity observed in 2016 (Figure 2B) likely resulted from reduced terrestrial fungi transport to stream ecosystems due to lower precipitation and water discharge, similar to findings by Arias-Real et al. (2021) under comparably arid conditions.

In contrast to the forested sites, the agricultural drainage ditch and mixed-use sites experienced changes shaped by anthropogenic activity. These changes may have been driven by leaching and runoff of agrochemicals and the application of livestock manure in the spring and fall, as observed within the watersheds of these sites (Wilkes et al., 2013; Sunohara et al., 2015), and rainfall-induced erosion of stream bank sediments (Frey et al., 2015). Such activities influenced the physicochemical properties of the sites, with nitrate levels and conductivity emerging as dominant factors associated with fungal alpha and beta diversity (Figures 2C, 3A). We found a positive correlation between water nitrate level and alpha diversity (Supplementary Figure S9), and nitrate also played a role in driving fungal beta diversity, consistent with previous studies. The enrichment of nitrogen in freshwater ecosystems has been linked to increased fungal bioactivity (Gulis et al., 2017), particularly noticeable in certain genera, such as *Betamyces*, which showed a positive correlation with nitrate levels at agricultural ditch and mixed-use sites (Figures 4A,B). Surprisingly, unlike other studies (Bai et al., 2018; Pietryczuk et al., 2018), pH did not show notable influences on stream mycobiota dynamics in our study, possibly due to minimal pH fluctuations across different land uses and throughout the study period (Figure 3A; Table 1).

Our study not only identified environmental drivers affecting the freshwater fungal community but also highlighted its potential resilience to fluctuations in water physicochemical properties. This resilience is evidenced by the relatively minor compositional changes over time at agricultural ditch and mixed-use sites, despite these locations experiencing greater water quality fluctuations compared to the forested site (Figure 3B). At the forested site, stronger correlations were observed between taxa abundance and water physicochemical properties, further corroborating this resilience (Figures 4A–C). For example, *Cladosporium* exhibited a strong positive correlation with nitrate at the forested site, where nitrate levels varied minimally, unlike at agricultural or mixed-use sites. This pattern of resilience mirrors findings in soil microbiomes by Chen Y. et al. (2022), where fungal communities showed less sensitivity to nutrient loads than bacterial communities. The freshwater fungal community may have undergone selective pressures that favor pollution-tolerant taxa, as seen in the Tietê river where Ortiz-Vera et al. (2018) observed fungal adaptation to elevated nitrogen and phosphorus levels from agricultural runoff. Such selective pressures at agricultural drainage ditch sites and mixed-use sites might explain the lower compositional changes and insensitivity to shifts in water quality, reflecting the presence of a narrower range of tolerant fungi in these environments.

Given the established influence of fungicides on fungal community structure and diversity (Staley et al., 2015; Ittner et al., 2018; Baudy et al., 2021; Ma et al., 2021), we anticipated that fungicide exposure would play a role in shaping the stream fungal communities. However, data from 2018–2020 showed that fungicides and their interaction with water physicochemical properties accounted for only 5% of the variation in fungal communities, while water properties alone explained over 20% of the variation during the same period. This may be attributed to the low fungicide concentrations observed in our study, which were significantly lower than the concentrations (>5 μg/L) known to strongly affect fungal communities (Baudy et al., 2021). A review by Zubrod et al. (2019) reported mean concentrations of Boscalid and Metalaxyl in North American studies to be 534 ng/L and 395 ng/L, respectively—both substantially higher than the levels detected in our study (Table 2). Fungicide levels in this study also did not show a strong correlation with precipitation or rainfall events, although rainfall erosion of sediments and soils can impact streams with little to no impact on discharge (Frey et al., 2015). Furthermore, CART analyses showed that fungicides had inconsistent effects on stream mycobiota diversity and the relative abundance of fungal genera, with some fungicides exhibiting positive correlations and others negative, but no conclusive pattern emerged (Supplementary Tables S6, S7). Overall, our results suggested that at the low fungicide concentrations encountered, stream fungal communities appeared relatively unaffected.

Our study also suggested that unknown or even counterintuitive factors may have influenced the stream mycobiota, especially over multiple sampling seasons. This was exemplified by the fact that water physicochemical properties explained 20% community variance during 2018–2020; yet these parameters accounted for less than 2.5% of the community variance over a longer period (2016–2021) (Figure 3A). This contrasts with earlier findings by Solé et al. (2008), who reported that these properties accounted for 86.2% of the variance in fungal community structure. The discrepancy may stem from methodological differences: Solé et al. (2008) examined a limited number of aquatic fungal species by examining conidia, whereas our study utilized metabarcoding approach to analyze whole communities. Additionally, the lower explained variance could be attributed to stochastic processes influencing community assembly, as seen in bacterial and fungal communities in wetland soils (Huang et al., 2022), alongside known environmental factors.

### 4.4 Functional resilience in stream fungal communities

Our study suggested that stream fungal communities may be functionally resilient to environmental stressors, such as inputs of agrochemicals. Despite changes in fungal diversity and community composition, the relative abundance of saprotrophic fungi showed no significant differences between agricultural drainage ditches, mixed-use, and forested sites (Figure 5). This resilience is consistent with findings from Pascoal and Cássio (2004) and Bruder et al. (2016), who found that nutrient enrichment and other stressors had minimal effects on leaf litter decomposition in agricultural streams, except in cases where reduced dissolved oxygen from fine sediment deposition hindered decomposition rates. We observed no variation in dissolved oxygen levels across land use classes (Table 1), which may explain the consistent saprotrophic functionality among the sites since the decomposition role of aquatic fungi is dependent on dissolved oxygen in freshwater streams (Medeiros et al., 2009). Furthermore, Baudy et al. (2021) showed that even under fungicide stress, stream fungal communities maintained their decomposition capacity, likely due to functional redundancy.

While our findings suggested that the saprotrophic role of fungi remained relatively unaffected across different land use classes, it is important to note that the FUNGuild (Nguyen et al., 2016; Furneaux, 2021) annotations used in this study were of lower resolution, with many assigned only at the genus or family level. This broader classification can limit the accuracy of functional predictions, as these categories may include diverse species with varying ecological roles, leading to potential redundancy in the functional assignments. Additionally, our study did not directly measure leaf litter decomposition rates, which would have provided a clearer indication of saprotrophic fungal activity in the stream. To better understand the impact of agricultural practices on the ecological roles of stream fungi, future research should include direct assessments of leaf litter decomposition using methods such as measuring dry leaf mass (Bruder et al., 2016) or analyzing gene expression related to litter decomposition via RNA sequencing (Bourne et al., 2020). These approaches would offer more direct insights into organic matter decomposition in these environments.

## 5 Conclusion

This study investigated the changes in the diversity, composition, and function of stream mycobiota within an agriculturally dominated water basin, focusing on the effects of different land uses in the upstream catchment areas, as well as agricultural activities and weather-induced changes in water physicochemical properties, fungicide levels, and hydrology.

- Classifying aquatic fungi remains challenging due to limited entries in aquatic fungal databases, hindering accurate identification of native aquatic fungal species from transient fungi and determining their ecological functions.
- Significant variations in steam mycobiota were observed across different land uses, with agricultural drainage ditches showing a marked reduction in alpha diversity compared to the undisturbed forested reference area.
- River discharge significantly influenced the diversity of stream mycobiota, likely as a result of runoff and drainage-induced factors mobilizing fungal species from terrestrial and aquatic sources.
- Water physicochemical properties, including fungicide levels, explained only a small portion of the variation in mycobiota communities. This suggests that stochastic processes or unidentified environmental factors played a more significant role in shaping these communities.
- Despite pressures from agricultural land use, the fungal communities demonstrated functional resilience. However, further research is necessary to evaluate how these agricultural practices affect crucial ecological processes, such as leaf litter decomposition in streams.

## Data Availability

The datasets presented in this study can be found in online repositories. The names of the repository/repositories and accession number(s) can be found at: https://www.ncbi.nlm.nih.gov/genbank/, PRJNA1018649.
