# Supplementary material for: The functions and factors governing fungal communities and diversity in agricultural waters: insights into the ecosystem services aquatic mycobiota provide
Source: Front Microbiol. 2024 Nov 5;15:1460330. doi: 10.3389/fmicb.2024.1460330 (PMC11574526; doi:10.3389/fmicb.2024.1460330)
Supplement: Supplementary file 1 [file Data_Sheet_1.pdf]

Supplementary Material

The functions and factors governing fungal communities and diversity in agricultural waters: insights into the ecosystem services aquatic mycobiota provide

Phillip Pham[1,2], Yichao Shi[1], Izhar Khan[1], Mark Sumarah[3], Justin Renaud[3], Mark Sunohara[1], Emilia Craiovan[1], David Lapen[1], Stéphane Aris-Brosou[2,4], Wen Chen[1,2*]

[1]Ottawa Research and Development Centre, Science and Technology Branch, Agriculture and Agri-Food Canada, Ottawa, ON, Canada

[2] Department of Biology, University of Ottawa, Ottawa, ON, Canada

[3]London Research Development Centre, Science and Technology Branch, Agriculture and Agri-Food Canada, London, ON, Canada

[4] Department of Mathematics and Statistics, University of Ottawa, Ottawa, ON, Canada

*** Correspondence:**
Wen Chen
wen.chen@agr.gc.ca

S.1 Supplemental Tables

Table S1. Sample summary by site, land use class, and year. Sampling periods:: May 24 – November 8 in 2016, May 1 – November 27 in 2017, April 30 – November 13 in 2018, April 29 – November 4 in 2019, July 13 – November 2 in 2020, and April 20 – November 8 in 2021.

Site ID [1]	Land Use	Number of Samples (n) [2]					
		2016	2017	2018	2019	2020	2021
5	Mixed	10	15	14	14	3	14
6	Mixed	12	15	14	14	3	14
10	Mixed	11	15	14	14	3	14
18	Agriculture	7	15	11	11	2	13
19	Agriculture	0	15	11	14	3	13
20	Agriculture	5	15	11	10	3	12
21	Agriculture	0	15	9	9	3	13
24	Forest	11	15	14	14	3	13

[1] Sampling at sites SN_19 and SN_21 started in the 2017 sampling season.
[2] Year 2020 was under sampled due to restrictions related to COVID-19 lockdowns.

Table S2. Definitions for land use, water physicochemical, and environmental variables in this study.

Variable name	Variable description (unit)
site_type	Sampling site land use class (Agriculture, Mixed, Forest)
strahler	Strahler stream order of the sampling site
TEMP_C	Temperature read with the YSI mini sonde in water (°C)
PH	pH read with the YSI mini sonde in water
CONDUCTIVITY_MSC	Water specific conductivity read with YSI mini sonde in water ($\mu S\ cm^{-1}$)
DISS_OXYGEN_MGL	Dissolved oxygen read with YSI mini sonde in water ($mg\ L^{-1}$)
ORP_MV	Electronic measurement of oxidation-reduction potential (mV)
TURBIDITY_NTU	Turbidity measurement with YSI mini sonde in Nephelometric Turbidity Units (NTU)
AMIA_AMN	NH_3, NH_4^+ concentration ($mg\ L^{-1}$)
NITRITE	Nitrite concentration ($mg\ L^{-1}$)
NITRATE	Nitrate concentration ($mg\ L^{-1}$)
TOTKN	Total Kjeldahl Nitrogen ($mg\ L^{-1}$)
TOTPHO	Total phosphorus concentration ($mg\ L^{-1}$)
DOC	Dissolved organic carbon concentration ($mg\ L^{-1}$)
TOC	Total organic carbon concentration ($mg\ L^{-1}$)
D_REA_PHOS	Dissolved reactive phosphorus concentration ($mg\ L^{-1}$)
RU_DISM3S	Discharge of Castor River at Russell station (02LB006), daily mean water discharge ($m^3\ s^{-1}$)
AG_nK_2014	Percentage of area covered by agricultural land use in basin up to n km upstream from the site. n = 2, 5, 10, 20 (%)
DEVELP_nK_2014	Percentage of area covered by developed land use in basin up to n km upstream from the site. n = 2, 5, 10, 20 (%)
TREE_nK_2014	Percentage of area covered by tree/forest land use in basin up to n km upstream from the site. n = 2, 5, 10, 20 (%)
WATER_nK_2014	Percentage of area covered by water in basin up to n km upstream from the site. n = 2, 5, 10, 20 (%)
OTHER_nK_2014	Percentage of area covered by all other undescribed land uses in basin up to n km upstream from the site. n = 2, 5, 10, 20 (%)
rain_mm_nd	Total rainfall recorded at WEBs meteorological station over n days prior, including sampling date. n = 1, 2, 3, 5, 7. (mm)
avg_temp_c_nd	Average temperature recorded at WEBs meteorological station over n days prior, including sampling date. n = 1, 2, 3, 5, 7. (°C)

Table S3. Summary of land use features at each sampling site. Land use percentages are reported as the percent land use in water catchment areas up to 5 km upstream from each sampling site.

Site ID	Land Use	Strahler stream order	Agriculture (% basin)	Developed (% basin)	Tree (% basin)	Other [1] (% basin)
SN_5	Mixed	4	89.91	1.83	6.39	1.87
SN_6	Mixed	5	94.09	0	4.09	1.82
SN_10	Mixed	4	77.75	20.05	1.93	0.27
SN_18	Agriculture	2	99.07	0	1.93	0
SN_19	Agriculture	2	100.00	0	0	0
SN_20	Agriculture	2	98.41	0	1.56	0.02
SN_21	Agriculture	2	96.73	0	3.27	0
SN_24	Forest	1	2.13	0	97.87	0

[1] Other land use category consists of grassland, wetland, and rock covered area.

Table S4. Method performance parameters for target fungicide analytes.

Substance	Ion Type	RT (min)	Precursor (m/z)	Quantifier m/z (CE)	Qualifier m/z (CE)	MDL / MQL (μg/L)	IS/SUR
Azoxystrobin	[M+H]+	13.76	404.124	371.93 (15)	328.86 (31)	0.00028/0.001	IS-1
Boscalid	[M+H]+	13.88	343.04	307 (20)	271.11 (33)	0.00345/0.01	Ext
Difenoconazole	[M+H]+	15.39	406.071	250.85 (26)	336.85 (18)	0.0003/0.001	IS-2
Metalaxyl	[M+H]+	11.28	280.154	220.05 (14)	248.05 (10)	0.00078/0.001	IS-3
Picoxystrobin	[M+H]+	15.64	368.11	145.05 (22)	205.07 (10)	0.0003/0.001	IS-4
Propiconazole	[M+H]+	14.67	342.077	159 (30)	122.95 (54)	0.00028/0.001	IS-2
Pyraclostrobin	[M+H]+	16.12	388.105	193.9 (13)	162.98 (24)	0.0003/0.001	IS-4
Spiroxamine	[M+H]+	11.52	298.274	144.05 (21)	99.93 (31)	0.00028/0.001	Ext
Tebuconazole	[M+H]+	13.99	308.152	69.97 (23)	124.9 (37)	0.00028/0.001	IS-5
Trifloxystrobin	[M+H]+	17	409.136	185.917 (17)	144.917 (43)	0.00028/0.001	IS-4
Thiabendazole	[M+H]+	3.13	202.043	174.97 (26)	130.97 (33)	0.00028/0.001	IS-6
Azoxystrobin-d4	[M+H]+	13.73	408.15	376.08 (14)			
Propiconazole-D7	[M+H]+	14.64	349.12	158.917 (29)			
Metalaxyl-D6	[M+H]+	11.23	286.191	226.054 (14)			
Pyraclostrobin-D6	[M+H]+	16.07	394.105	200.083 (12)			
Tebuconazole-D9	[M+H]+	13.94	317.208	69.97 (23)			
Thiabendazole-D4	[M+H]+	3.07	206.07	179 (25)			

Table S5. Summary of the sequencing reads and OTUs for each fungal phylum.

**Phylum**	**Recovered Sequences**	**Proportion of Recovered Sequences (%)**	**Number of OTUs**	**Proportion of OTUs (%)**
Ascomycota	8,703,053	86.85	4,859	73.95
Basidiomycota	854,365	8.53	1,219	18.55
Chytridiomycota	426,997	4.26	333	5.07
Rozellomycota	10,860	0.11	41	0.62
Aphelidiomycota	9,108	0.09	18	0.27
Olpidiomycota	6,381	0.06	10	0.15
Mortierellomycota	4,314	0.04	32	0.49
Mucoromycota	2,353	0.02	21	0.32
Monoblepharomycota	2,307	0.02	13	0.20
Blastocladiomycota	788	0.01	10	0.15
Basidiobolomycota	445	< 0.01	6	0.09
Glomeromycota	169	< 0.01	4	0.06
GS01	26	< 0.01	2	0.03
Kickxellomycota	21	< 0.01	1	0.02
Entomophthoromycota	15	< 0.01	1	0.02
Entorrhizomycota	11	< 0.01	1	0.02

Table S6. Two-node CART trees where stream mycobiota diversity value was the dependent variable and fungicides were the independent variables (bold indicates greater mean relative abundance).

**Diversity Index**	**Mean±SD**	**N**	R^2	**Splitter Fungicide**	**Fungicide Split Criteria (ng L^{-1})**	**Diversity Index When Less Than or Equal to Split Criteria**		**Diversity Index When Greater Than Split Criteria**	
						**Mean±SD**	**N**	**Mean±SD**	**N**
Shannon TD	23.0±14.4	93	0.059	Propoconazole	2.5	**24.0±14.5**	86	10.8±3.5	7
Simpson TD	10.8±7.6	93	0.143	Difenoconazole	1.0	9.0±7.3	49	**12.7±7.3**	44

Table S7. Two-node CART trees where relative abundance of a given fungal genus was the dependent variable and fungicides were the independent variables (bold indicates greater mean relative abundance).

Fungal Genera Relative Abundance			R^2	Splitter Fungicide	Fungicide Split Criteria (ng L^{-1})	Relative Abundance When Less Than or Equal to Split Criteria		Relative Abundance When Greater Than Split Criteria	
	**Mean±SD**	**N**				**Mean±SD**	**N**	**Mean±SD**	**N**
Ramularia	3.2±7.4	93	0.148	Metalaxyl	1.9	**8.3±10.8**	22	1.6±5	71
Betamyces	2.6±6.5	93	0.484	Difenoconazole	1.0	**4.2±8.6**	49	0.8±1.3	44
Sphaerulina	3.6±9.7	93	0.259	Tebuconazole	10.0	1.8±6.1	82	**17.0±17.0**	44
Neosetophoma	0.8±1.2	93	0.131	Metalaxyl	9.4	0.4±0.7	55	**1.3±1.5**	38
Peniophora	0.05±0.14	93	0.215	Picoxystrobin	0.8	0.03±0.08	84	**0.26±0.32**	9
Venturiocistella	0.01±0.07	93	0.903	Pyracolstrobin	10.9	0.001±0.012	77	**0.054±0.166**	16

S.2 Supplementary Figures

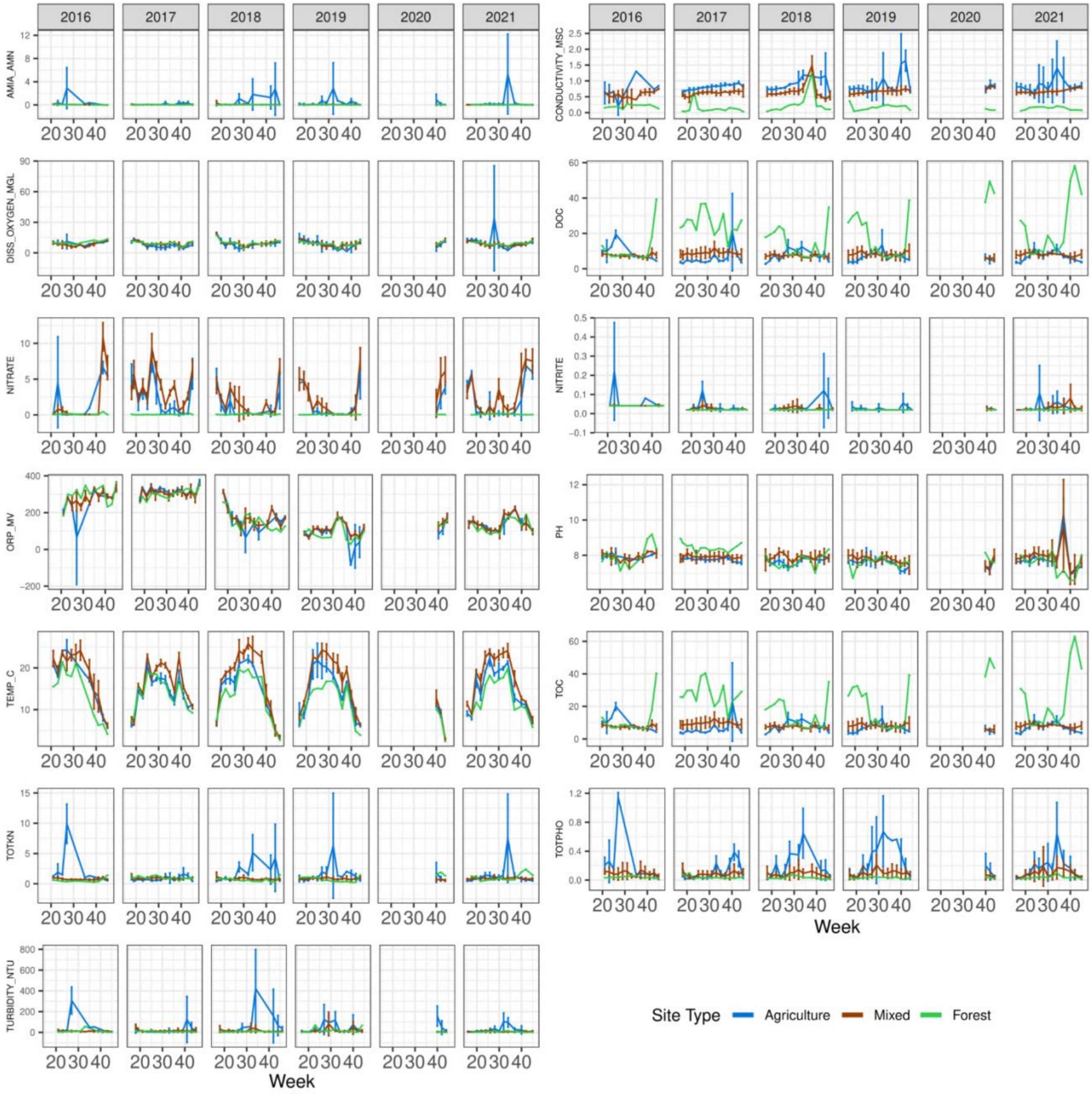

Figure S1. Temporal variation of water physicochemical properties across land use classes. Error bars represent the standard error around the data points. No error bars are reported for forested sites due to n = 1 replicate per time point. Variable descriptions for water physicochemical properties are listed in Supplementary Table S2.

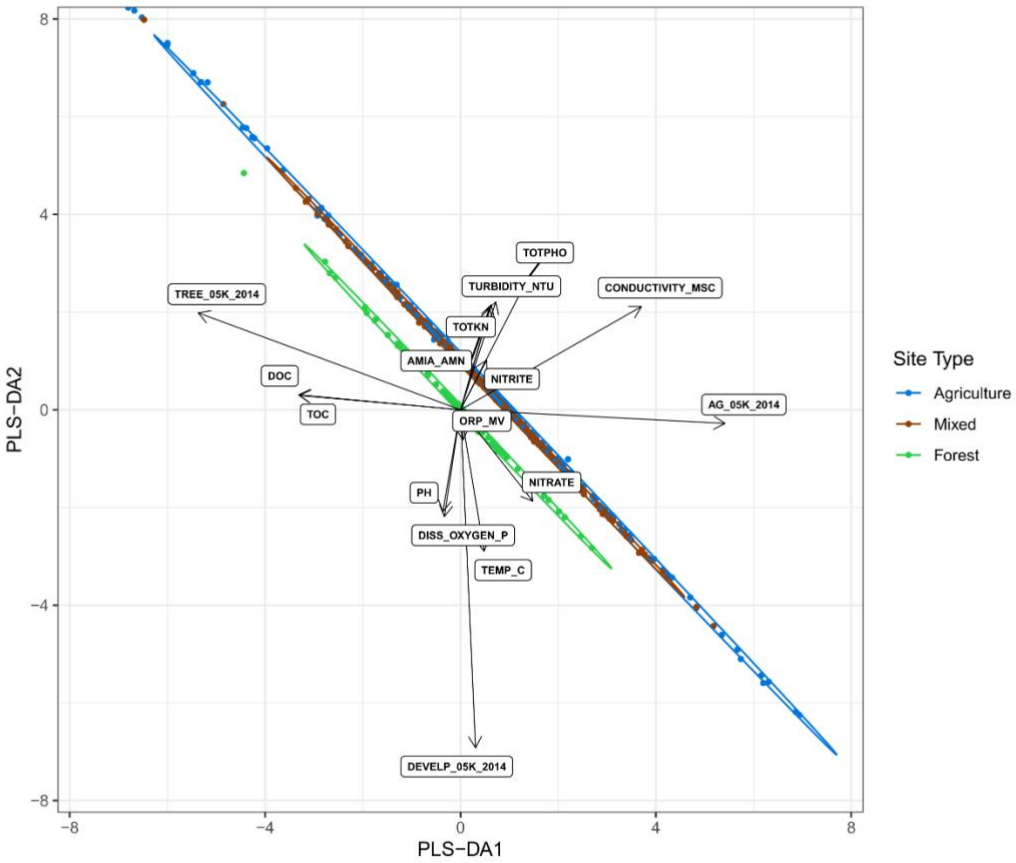

Original	Predicted		
	Agriculture	Mixed	Forest
Agriculture	115	105	0
Mixed	94	119	0
Forest	8	15	47

Figure S2. PLS-DA of sampling sites based on water physicochemical and land use variables, with the classification confusion matrix for land use classes. The overall classification error rate is high (0.44) due to the similarity in water physicochemical properties between mixed-use and agricultural ditch sites, both primarily influenced by agricultural activities.

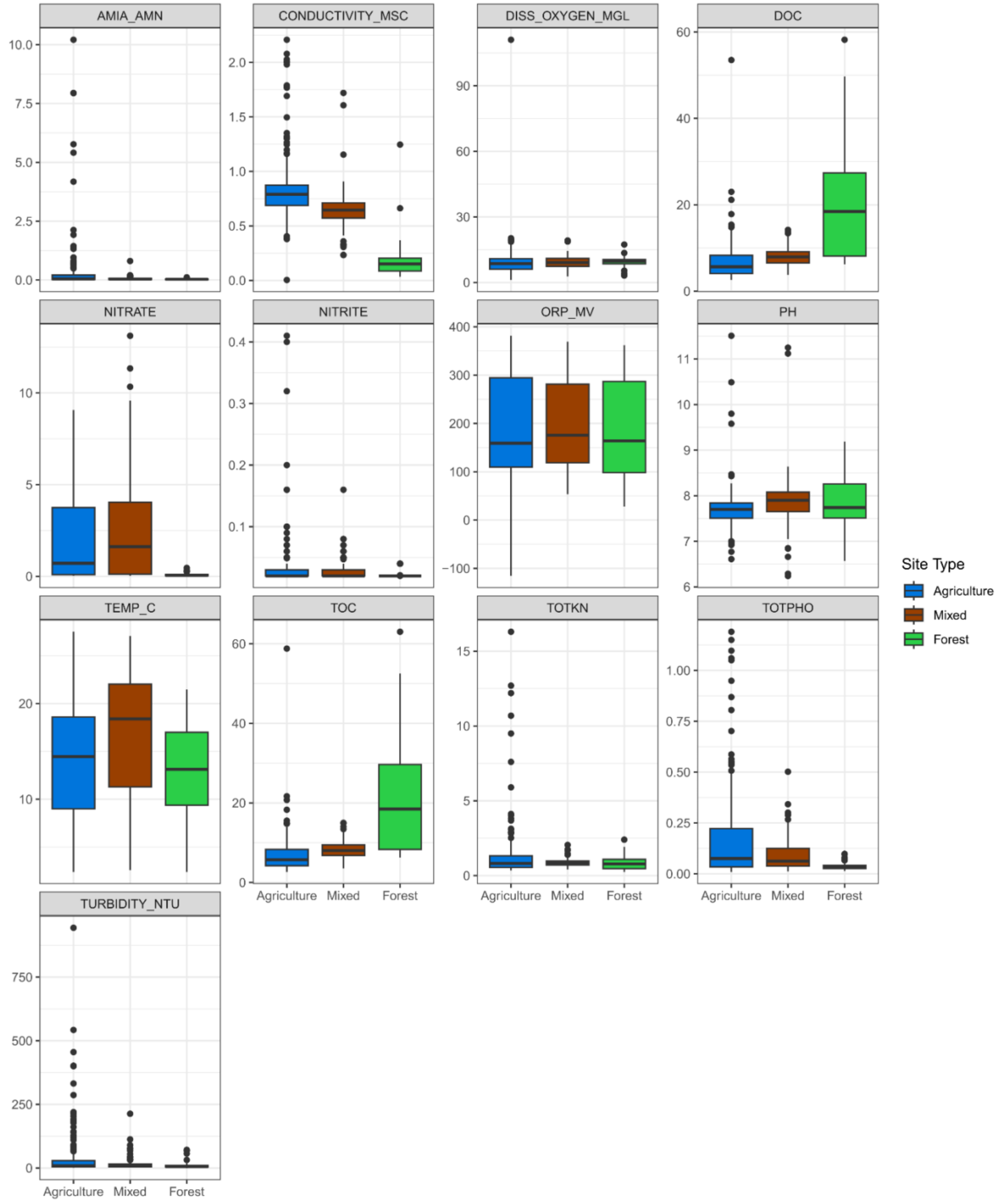

Figure S3. Boxplots of stream water physicochemical properties grouped by land use class.

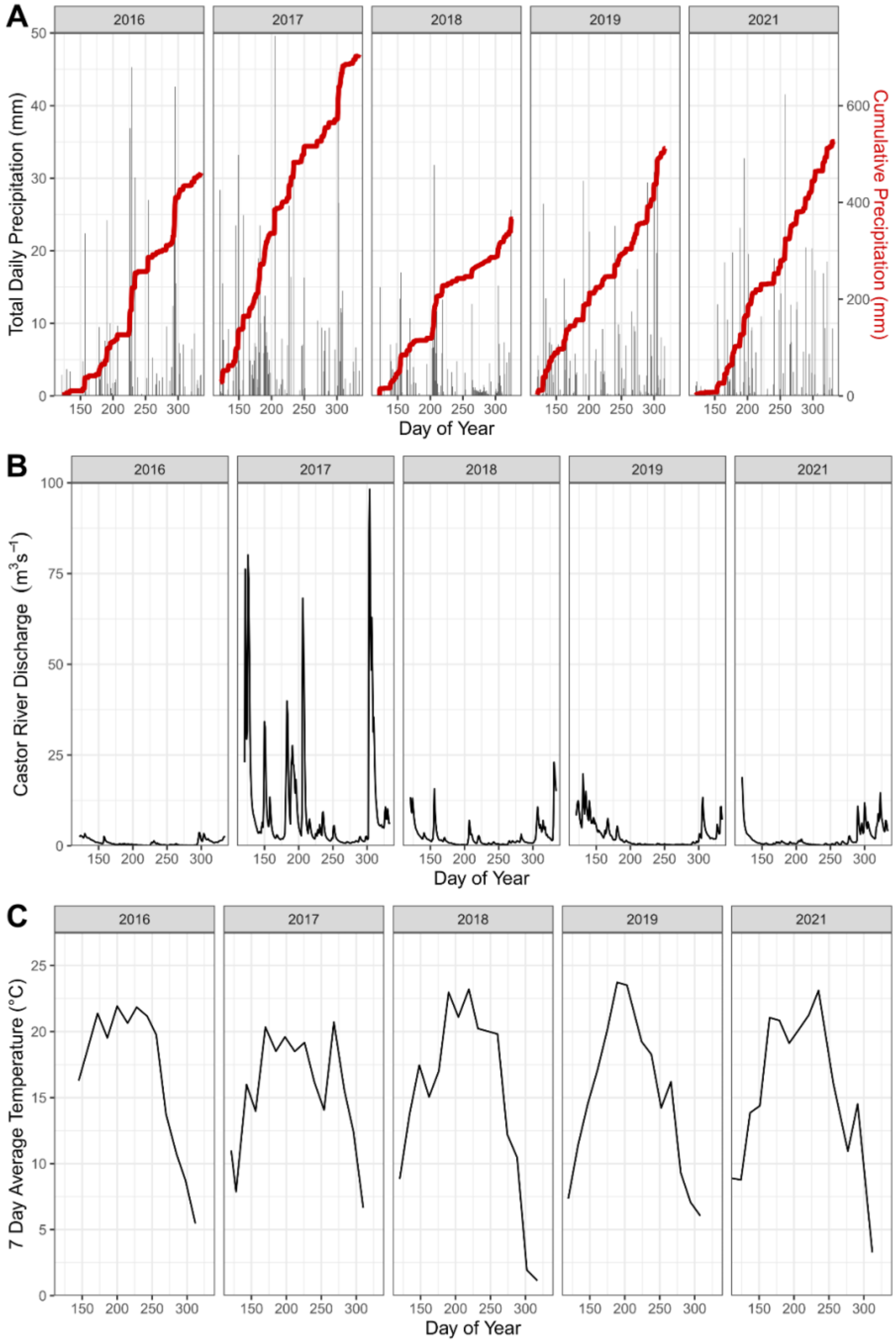

Figure S4. Weather conditions in the South Nation River basin. (A) Daily cumulative precipitation during the sampling season. (B) Daily mean water discharge of the Castor River, measured at Russell station (02LB006). (C) Mean temperature averaged over 7 days prior to the sampling date. Data were collected during the sampling season, ranging from days 110 – 317 of the calendar year (May – November). Year 2020 was not plotted due to under sampling during the COVID-19 lockdowns.

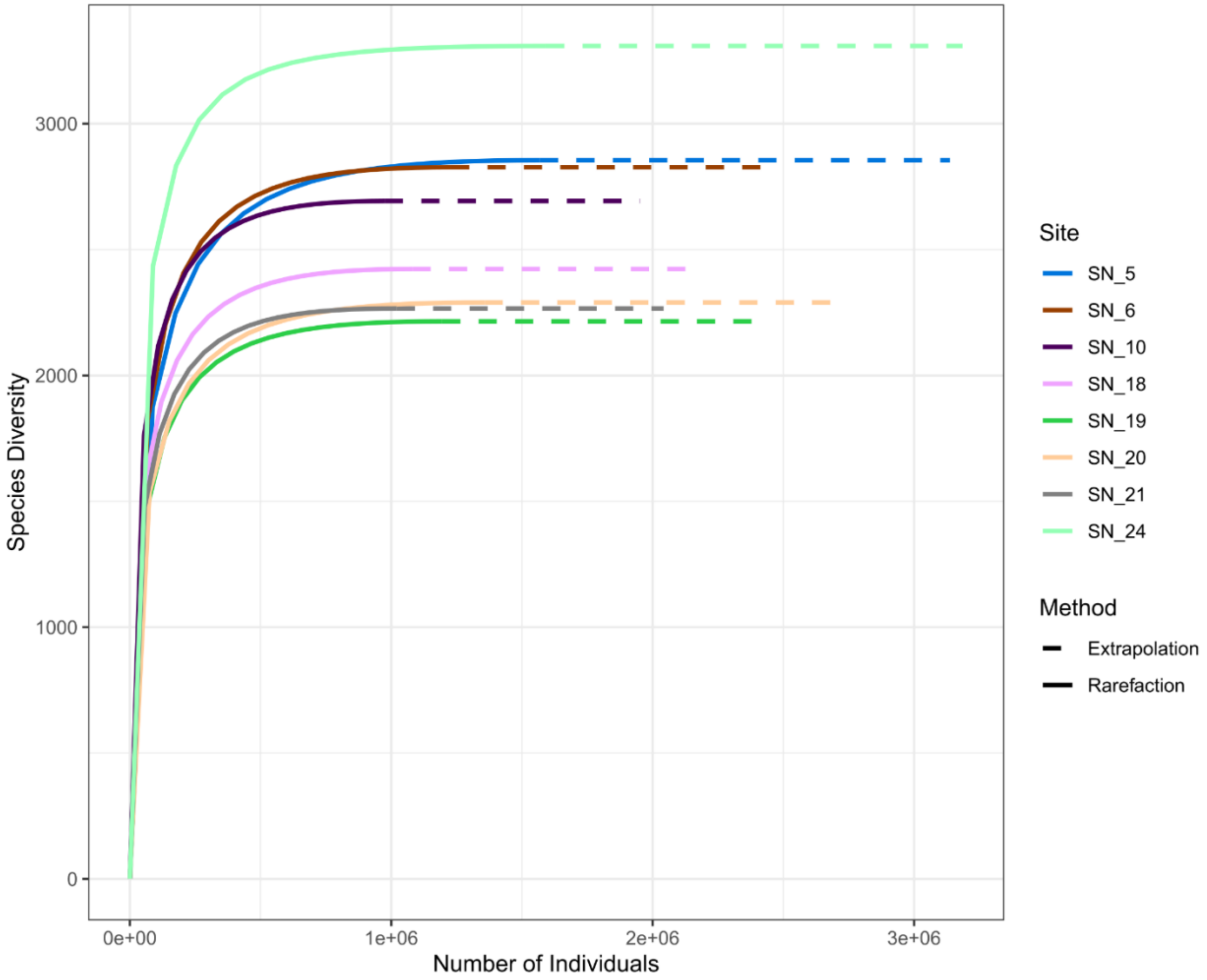

Figure S5. Rarefaction curves of OTUs obtained at each site, with extrapolation curves predicted using the iNEXT package. The horizontal asymptotes of the rarefaction curves indicate that saturated sampling was achieved at each site.

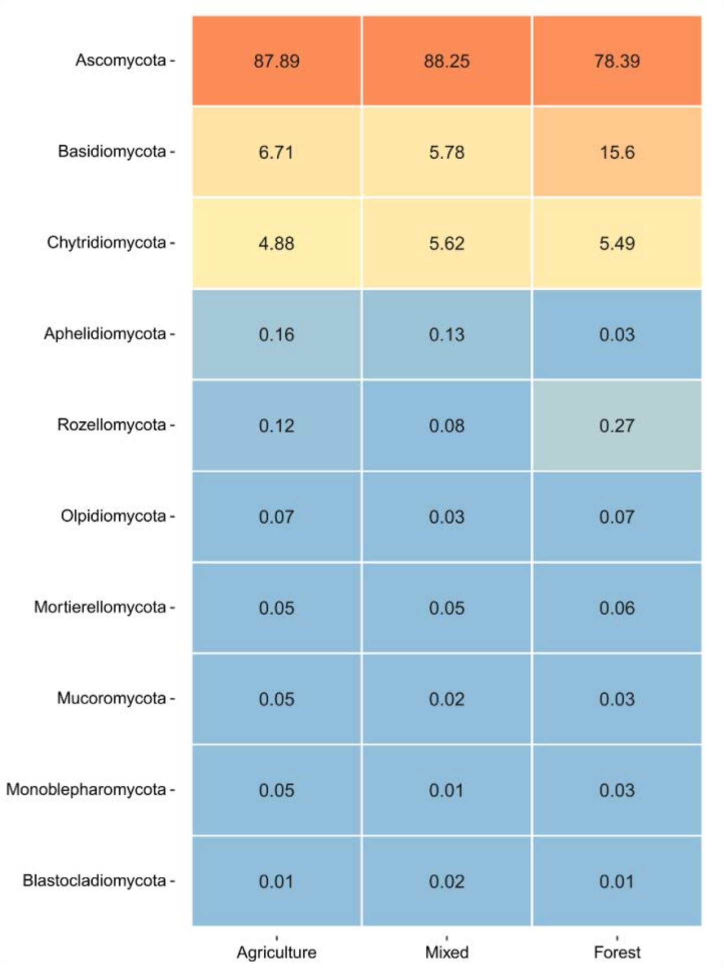

Figure S6. Mean relative abundance of fungal phyla by land use classes. Differences in mean relative abundance were computed using linear mixed-effects modeling, with land use class as a categorical fixed effect, and sampling date and sampling block as random effects. Pairwise comparisons were performed using estimated marginal means with Tukey P value adjustment. Ascomycota abundance was greater at agricultural and mixed-use sites compared to the forested site (agr-forest, $P = 0.0219$; mixed-forest, $P = 0.0184$), while Basidiomycota abundance was greater at the forested site (agr-forest, $P = 0.0082$; mixed-forest, $P = 0.0058$).

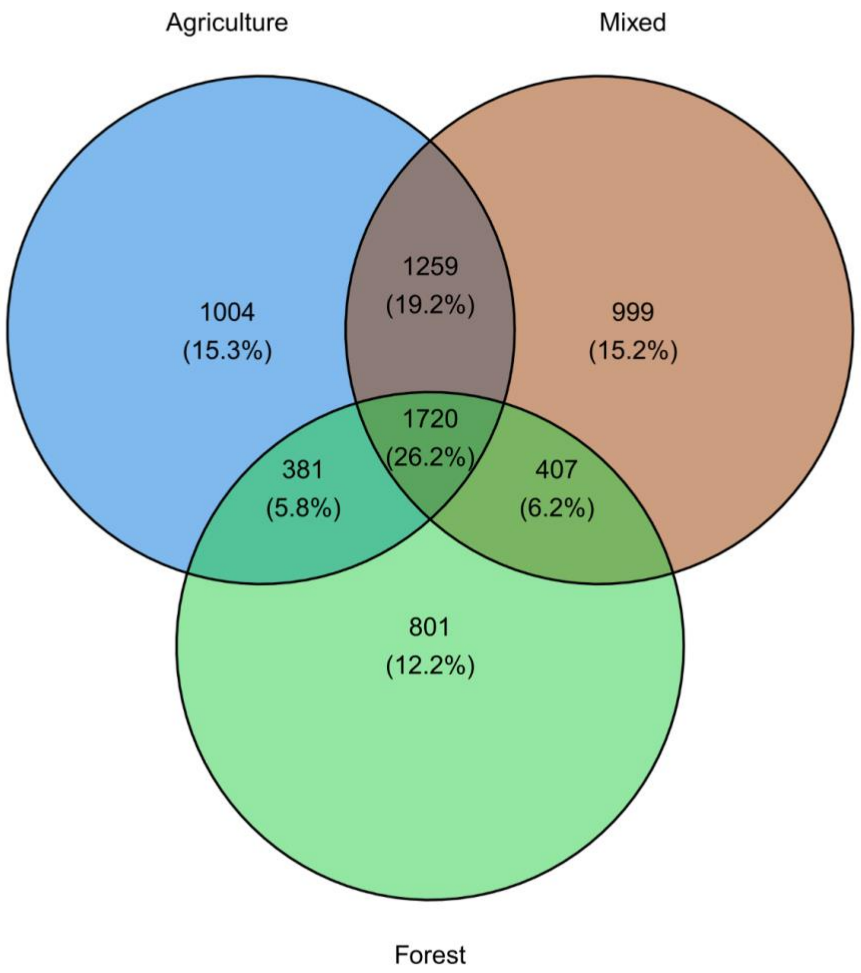

Figure S7. Venn digram showing the number of unique and shared OTUs across different land use classes.

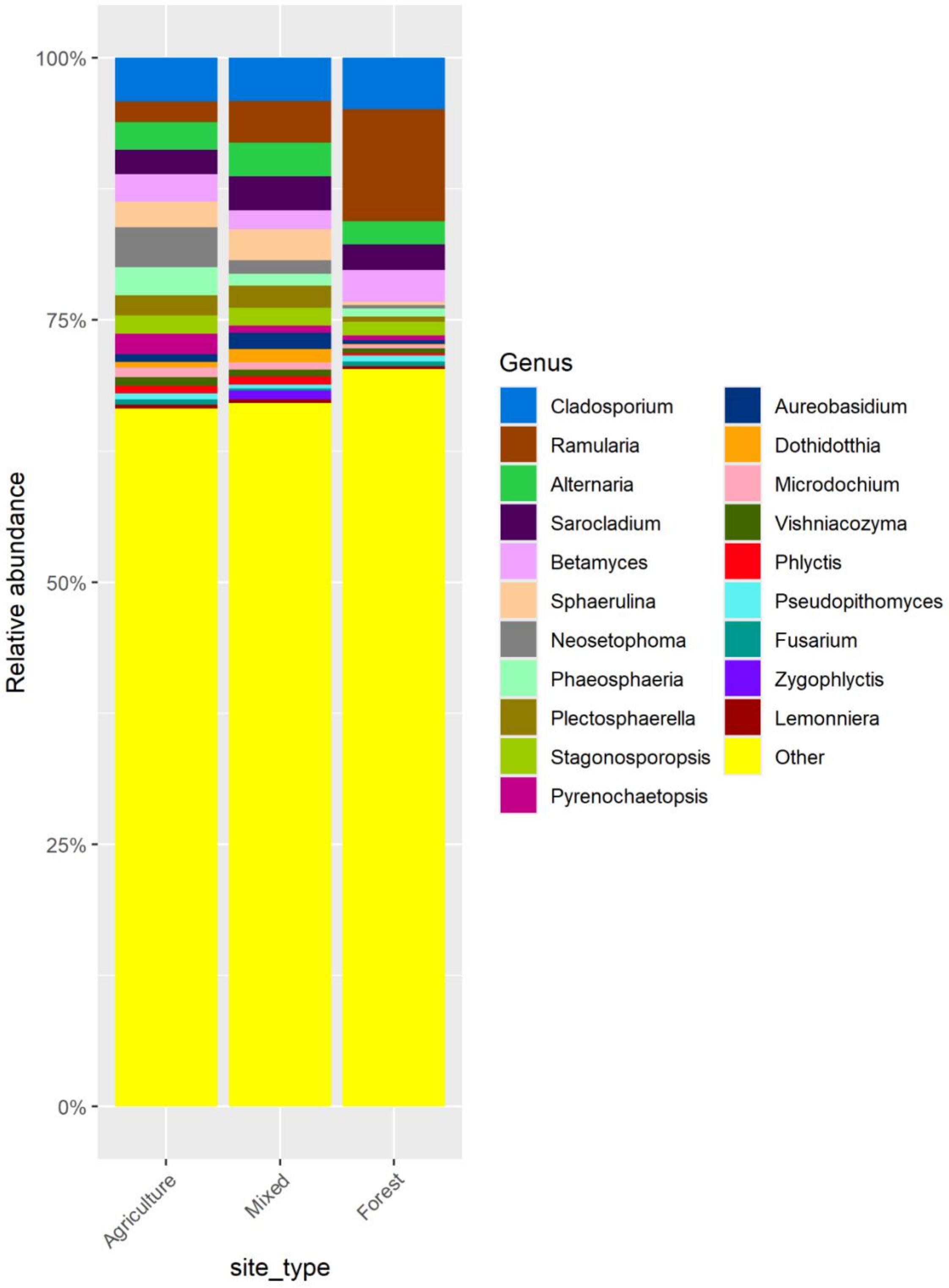

Figure S8. Mean relative abundance of the top 20 most abundant freshwater fungal genera across different land use classes.

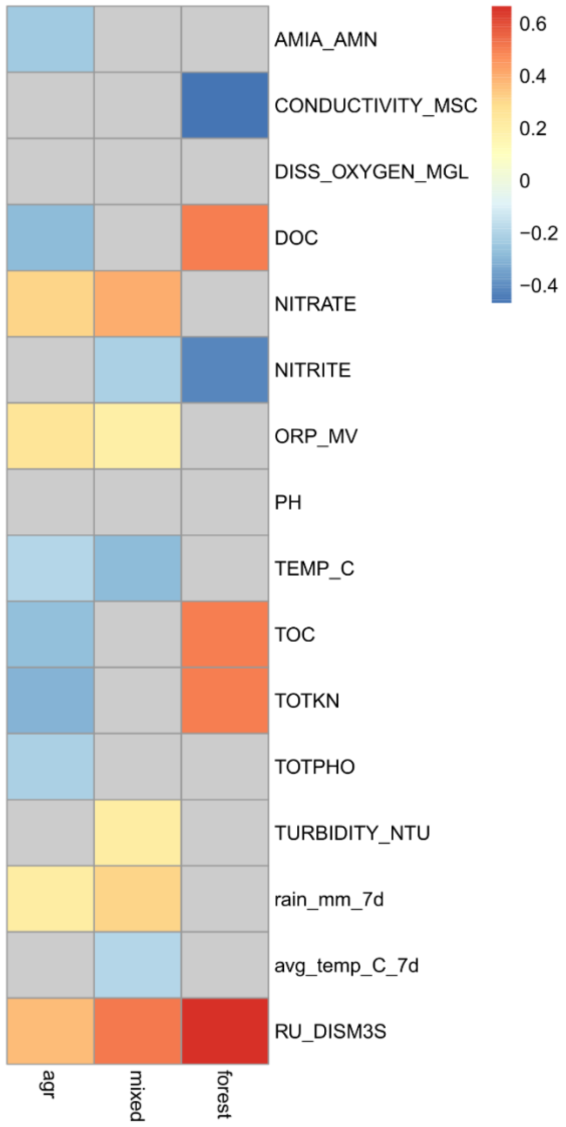

Figure S9. Correlation between alpha diversity and water physicochemical properties for each land use class. Coloured cells indicate significant correlation at a significance level of $P < 0.05$.

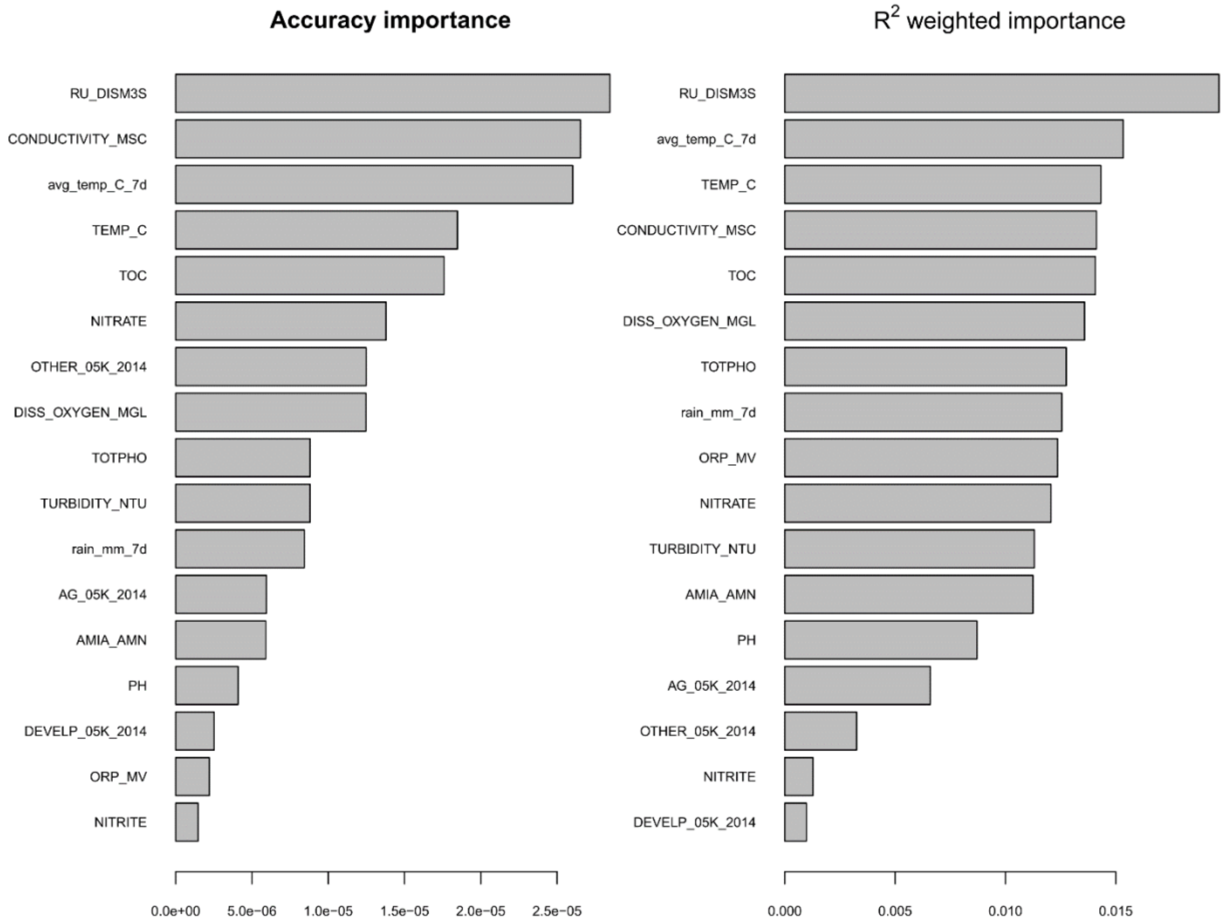

Figure S10. The most important environmental variables influencing stream mycobiota community variance, based on the Gradient Forest algorithm.

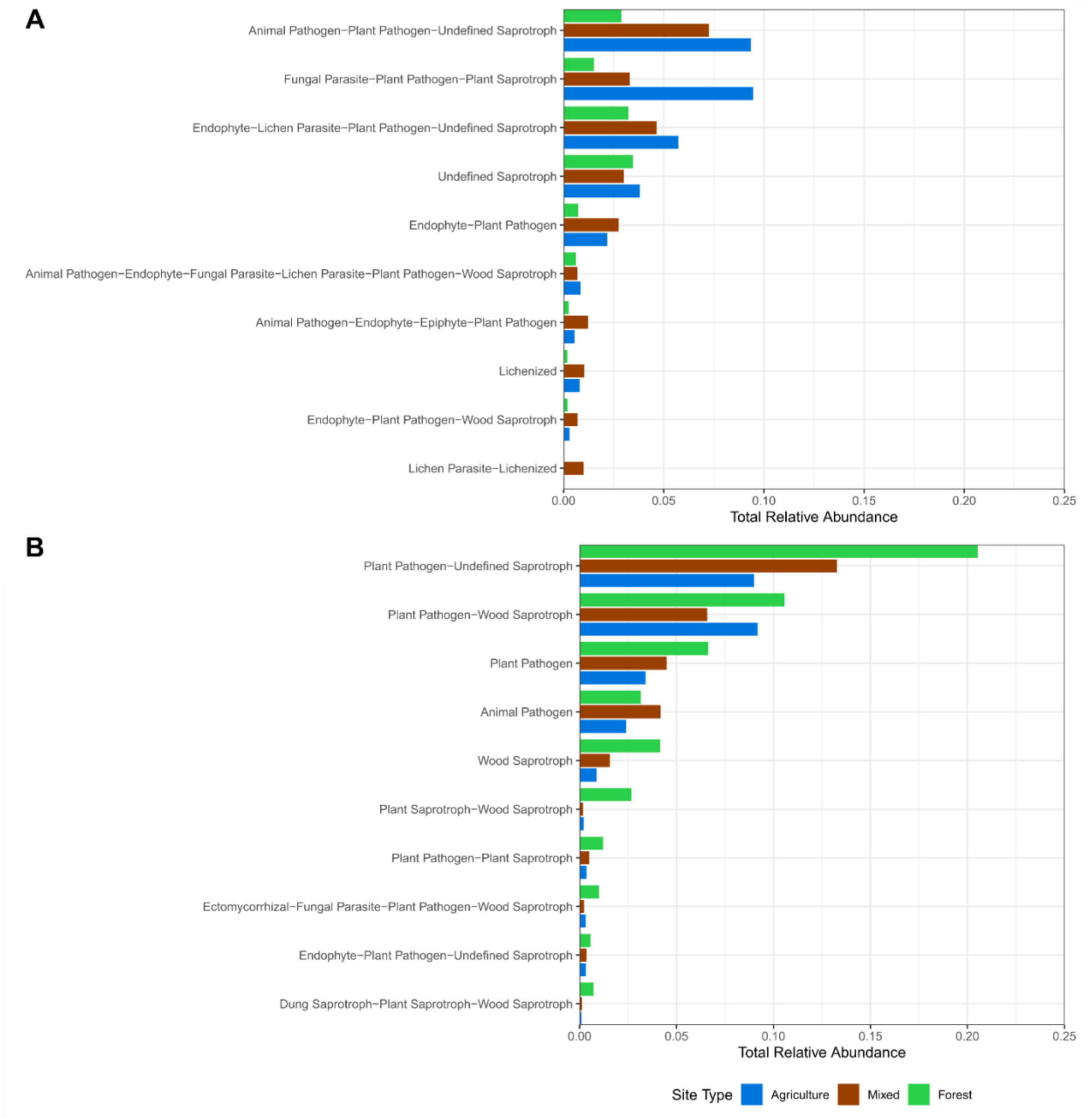

Figure S8. Relative abundance of predominant fungal functional guilds across different land use classes. (A) Guilds with higher relative abundance in agricultural sites compared to the forested site. (B) Guilds with higher relative abundance in the forested site compared to agricultural sites.

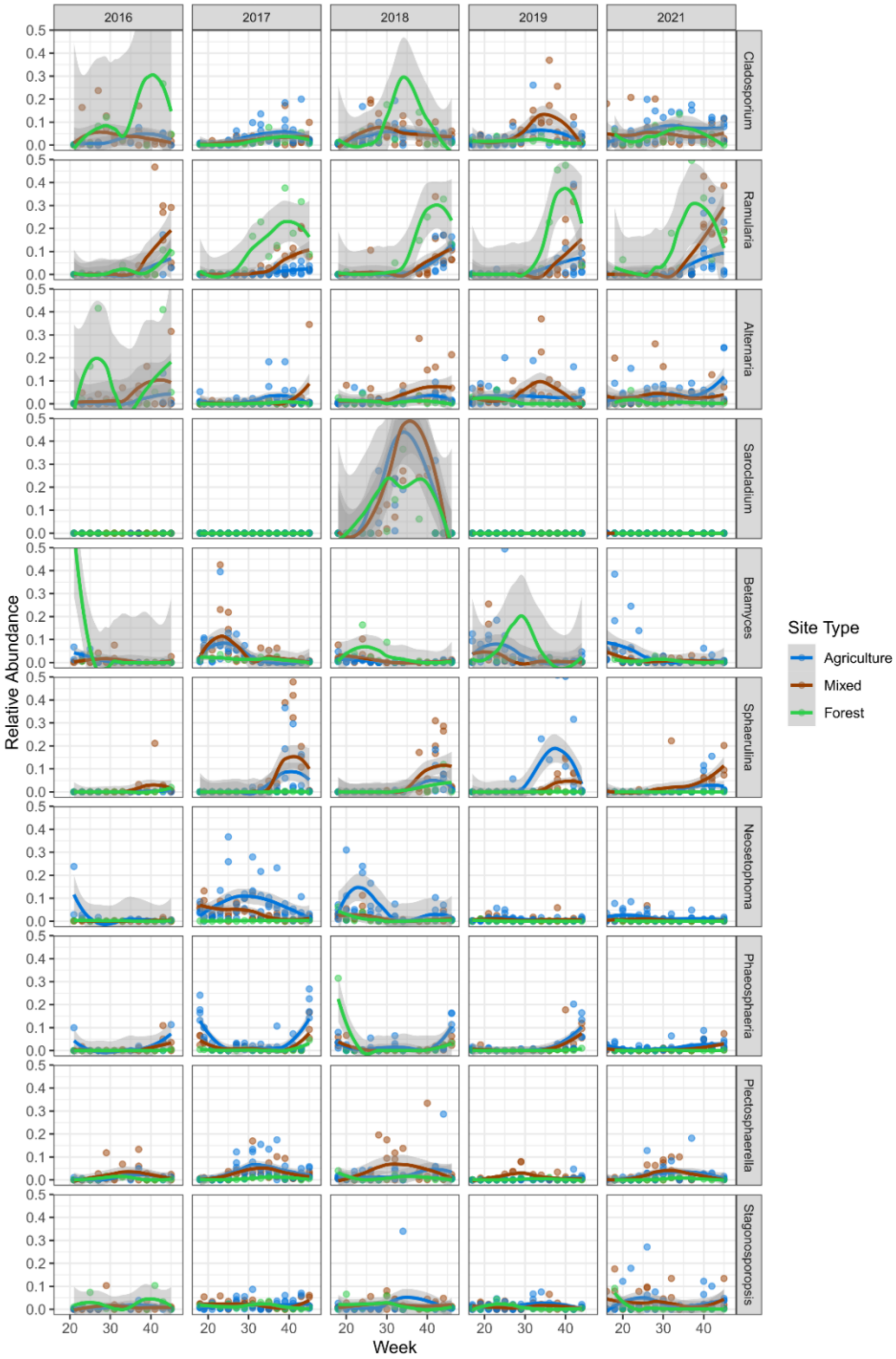

Figure S9. Temporal variation in the relative abundance of the top 10 most prevalent genera across all land use classes. The year 2020 was excluded due to limited sampling during the COVID-19 lockdowns.
